# Microbial Communities and Interactions of Nitrogen Oxides With Methanogenesis in Diverse Peatlands of the Amazon Basin

**DOI:** 10.3389/fmicb.2021.659079

**Published:** 2021-06-29

**Authors:** Steffen Buessecker, Zacary Zamora, Analissa F. Sarno, Damien Robert Finn, Alison M. Hoyt, Joost van Haren, Jose D. Urquiza Muñoz, Hinsby Cadillo-Quiroz

**Affiliations:** ^1^School of Life Sciences, Arizona State University, Tempe, AZ, United States; ^2^Department of Biogeochemical Processes, Max Planck Institute for Biogeochemistry, Jena, Germany; ^3^Biosphere 2 Institute, University of Arizona, Oracle, AZ, United States; ^4^Honors College, University of Arizona, Tucson, AZ, United States; ^5^Laboratory of Soil Research, Research Institute of Amazonia’s Natural Resources, National University of the Peruvian Amazon, Iquitos, Peru; ^6^School of Forestry, National University of the Peruvian Amazon, Iquitos, Peru; ^7^Swette Center for Environmental Biotechnology, The Biodesign Institute, Arizona State University, Tempe, AZ, United States; ^8^Center for Fundamental and Applied Microbiomics, The Biodesign Institute, Arizona State University, Tempe, AZ, United States

**Keywords:** Amazon peatlands, nitrogen oxides, peat geochemistry, methanogens, microbial communities and interactions

## Abstract

Tropical peatlands are hotspots of methane (CH_4_) production but present high variation and emission uncertainties in the Amazon region. This is because the controlling factors of methane production in tropical peats are not yet well documented. Although inhibitory effects of nitrogen oxides (NO_*x*_) on methanogenic activity are known from pure culture studies, the role of NO_*x*_ in the methane cycling of peatlands remains unexplored. Here, we investigated the CH_4_ content, soil geochemistry and microbial communities along 1-m-soil profiles and assessed the effects of soil NO_*x*_ and nitrous oxide (N_2_O) on methanogenic abundance and activity in three peatlands of the Pastaza-Marañón foreland basin. The peatlands were distinct in pH, DOC, nitrate pore water concentrations, C/N ratios of shallow soils, redox potential, and ^13^C enrichment in dissolved inorganic carbon and CH_4_ pools, which are primarily contingent on H_2_-dependent methanogenesis. Molecular 16S rRNA and *mcrA* gene data revealed diverse and novel methanogens varying across sites. Importantly, we also observed a strong stratification in relative abundances of microbial groups involved in NO_*x*_ cycling, along with a concordant stratification of methanogens. The higher relative abundance of ammonia-oxidizing archaea (Thaumarchaeota) in acidic oligotrophic peat than ammonia-oxidizing bacteria (*Nitrospira*) is noteworthy as putative sources of NO_*x*_. Experiments testing the interaction of NO_*x*_ species and methanogenesis found that the latter showed differential sensitivity to nitrite (up to 85% reduction) and N_2_O (complete inhibition), which would act as an unaccounted CH_4_ control in these ecosystems. Overall, we present evidence of diverse peatlands likely differently affected by inhibitory effects of nitrogen species on methanogens as another contributor to variable CH_4_ fluxes.

## Introduction

Estimates of methane (CH_4_) emissions in the tropics have high uncertainties due to limited spatial and temporal *in-situ* monitoring and a poor mechanistic understanding of soil CH_4_ sources and sinks (Kirschke et al., 2013). Multiple studies have evaluated the mechanisms of CH_4_ flux in soils (Verchot et al., 2000; Blodau, 2002; Whalen, 2005; Elberling et al., 2011). However, spatial and temporal CH_4_ flux variability in most terrestrial ecosystems is high, particularly in the Amazon basin (Bloom et al., 2010; Davidson et al., 2012), and with low modeling predictability (Parker et al., 2018). Peatlands have been overlooked as strong CH_4_ sources within tropical latitudes (Sakabe et al., 2018; Tang et al., 2018a; Wong et al., 2018, 2020; Finn et al., 2020), including recently documented sites in the Peruvian Amazon (Teh et al., 2017; Winton et al., 2017; Finn et al., 2020). Large swaths of areas holding tropical peatlands have been reported in the Amazon basin (Gumbricht et al., 2017). The Pastaza-Marañón foreland basin (PMFB) contains soil carbon stocks estimated to be at least 3.1 × 10^12^ kg, or ∼32% that of South America (Draper et al., 2014), which could be slightly underestimating true extents according to more recent predictions (Gumbricht et al., 2017). Therefore, peatlands of the PMFB pose a major potential source for atmospheric CH_4_ in the region. Given the role of CH_4_ as a powerful greenhouse gas and the predicted shift in environmental conditions of the Amazon basin in the wake of global climate change (Davidson et al., 2012), it is critical to address uncertainties in CH_4_ flux in these environments.

In water logged peat soils, oxygen (O_2_) depletion due to the imbalance between slow O_2_ diffusion in water and rapid O_2_ consumption by heterotrophic respiration (Elberling et al., 2011) remains a main regulator of methanogenesis (Conrad, 2020). However, the influence from other high-redox species, such as nitrogen oxides (NO_*x*_), are rarely assessed in some microbial soil habitats. NO_*x*_ species are a set of N-O compounds, including nitrate (NO_3_^–^), nitrite (NO_2_^–^), and nitric oxide (NO). The distribution of NO_*x*_ species and methanogenesis is commonly heterogenous, however, it has been frequently found that in peatlands vertical soil stratification under anoxic conditions dictates microbial composition (Jackson et al., 2008; Noll et al., 2005; Watanabe et al., 2010; Puglisi et al., 2014; Bai et al., 2017) as well as methanogenic activity (Cadillo-Quiroz et al., 2006), and also leads to the formation of NO_*x*_ gradients (Pett-Ridge et al., 2006; Senga et al., 2015; Stone et al., 2015; Wang et al., 2016). Importantly, the microbial metabolism for NO_*x*_ production and consumption has been found to change with depth in sub-tropical forest soil (Tang et al., 2018b). Within diverse microbial communities, fermentative enzymes and NO_*x*_ reductases have been shown to correlate with chemical gradients (Chen et al., 2017). In tropical soils, heterotrophic denitrification and dissimilatory nitrate reduction to ammonium (DNRA)—a competitive reductive reaction to denitrification based on the common substrate NO_2_^–^—have been identified as the main pathways of NO_*x*_ cycling. Of these two pathways, denitrification (i.e., the reduction of NO_*x*_ to N_2_O and N_2_) is the dominant nitrogen loss pathway in tropical biomes, accounting for 24–53% of total ecosystem nitrogen loss (Hall and Matson, 1999; Houlton et al., 2006). Depending on the geochemical conditions (pH, DOM, metals) and microbial activity, NO_*x*_ are differently distributed in soils, which can affect the microbial community. For instance, NO_3_^–^ concentrations are typically higher in shallow soil layers where microbial nitrification is most active, but it can also accumulate in deeper layers, where its consumption may be inhibited by humic substances with phenol moieties (Senga et al., 2010). Meanwhile, NO_2_^–^ is unstable in acidic peat pore water, because it decomposes under acidic conditions (pH < 5.5) and rapidly reacts with organic soil moieties or metals (Cleemput and Samater, 1996). The relative degree to which soil microbiota are exposed to NO_2_^–^ is controlled by the inherent soil properties in addition to the activity of microbial NO_2_^–^ oxidoreductases or reductases. Their variable availability and interactions make NO_*x*_ generally difficult to identify as stimulators or inhibitors of microbial processes. Effects of NO_*x*_ have to be evaluated within the geochemical context of their soils or complementarily tested under controlled conditions.

Methanogens have been shown to be inhibited by free NO_*x*_ in pure culture experiments (Klüber and Conrad, 1998b) and paddy soil incubations (Klüber and Conrad, 1998a). In addition, NO_*x*_ can also serve as oxidants in the anaerobic oxidation of CH_4_ (AOM). In contrast to marine taxa that rely on sulfate, members of both the domains *Archaea* and *Bacteria* possess the potential to use NO_3_^–^ (Haroon et al., 2013) and NO_2_^–^ (Ettwig et al., 2010) to oxidize CH_4_. The metabolism of AOM-mediating bacteria (known as NC-10, and affiliated *Candidatus* Methylomirabilis oxyfera) has been demonstrated, and members of this clade were detected in sub-tropical peat soil (Hu et al., 2014). Thus, the concentration and species make-up of NO_*x*_ have the potential to influence the production and flux of CH_4_ in anaerobic soils, such as those in tropical peatlands.

In this study, we focused on three peatlands in the western Peruvian Amazon, locally known as *San Jorge* (SJO), *Buena Vista* (BVA), and *Quistococha* (QUI), with divergent formation history (Lähteenoja et al., 2009) and representing the geochemical diversity of anoxic, tropical peat soils found in the PMFB (Supplementary Figures 1, 2). Our goal was to evaluate the potential impact of nitrogen oxides on methane formation in geochemically diverse peatlands of the Amazon basin. We evaluated carbon and nitrogen pools, and the overall microbial community composition along soil profiles before we characterized the CH_4_ cycling based on isotopic signatures, and correlated methanogen abundances with soil NO_*x*_ distribution. We complemented field efforts with site specific anoxic soil slurries and enrichment cultures to test effects of NO_*x*_ compounds on methanogenic activity. Our work provides evidence for an important role of diverse geochemical backgrounds and putative N and C cycling interactions affecting methanogenesis in Amazon peatlands.

## Materials and Methods

### Study Sites and Field Sampling

The studied peatlands are located in the Pastaza-Marañón foreland basin (PMFB), Loreto Region, in the Western Peruvian Amazon. *San Jorge* (SJO; 4°03′41.6″ S, 73°11′48.1″ W) is a dome-type acidic (pH ∼3.5) oligotrophic peatland characterized previously (Lähteenoja et al., 2009), *Quistococha* (QUI), located adjacent to a lagoon (3°50′5.6″ S, 73°19′24.0″ W) with a relatively high density of *Mauritia flexuosa* palms, is classified by vegetation as a palm swamp site with mildly acidic (pH ∼4.3) and poor to intermediate nutrients, and *Buena Vista* (BVA; 4°14′23.3″ S, 73°12′08.2″ W) is a minerotrophic near circumneutral (pH ∼6.5) nutrient rich site undergoing large riverine flood pulses reaching 5–6 meter high lasting several months (4–6) and vegetated by primary rich forest. Field work was conducted in Summer 2014 (QUI, SJO) and 2015 (BVA) after onset of the rain season when the water tables were at or close to the surface (QUI, BVA) or at ∼10 cm below soil surface (SJO). Samples for isotopic analyses were collected in 2017.

To extract soil pore water from a vertical profile of 1 m depth, PTFE tubing ending in a porous teflon macro-rhizon (4.5 mm OD, 0.15 μm pore size, Sunvalley Solutions) was pushed into the peat soil. Peat pore water was pulled with a syringe and filtered (0.8/0.2 μm pore size, Acrodisc) into acid-washed HDPE plastic bottles (Nalgene Nunc Int.). Samples for ion chromatography and spectrometric analysis received the biocide Thymol (Thermo Fisher Scientific) to a 100 mg/L final concentration (Cape et al., 2001). To sample soil gas for the quantification of CH_4_ and CO_2_, we used a modification to a soil gas equilibration-by-diffusion through gas-permeable teflon membrane method (Hu et al., 2014). Ten PTFE tubes of 10–100 cm length with a 10 cm-long teflon stub were inserted into the soil over an area of ∼1 m^2^, closed at the top, and left at place for 24 h. After the equilibration period, a gas-tight syringe (Monoject) was used to draw sample gas and to inject it into pre-evacuated glass vials through a butyl rubber stopper. All liquid and gas samples were stored under cold or frozen conditions during transport and upon analysis. To sample soil for DNA extraction, a Russian soil corer was used to take 1 m cores. Three samples of 500 mg soil were aseptically weighted into screw cap tubes at every 10 cm, for two soil cores at each site (60 samples per site). Soil samples were kept frozen throughout the field work and transport. A 1:5, soil:water slurry was prepared and measured with a pH meter 10A (Ecosense, YSI), using the same soil depth intervals as for molecular work.

### Geochemical Analyses and Determination of the Carbon Isotopic Composition

Pore water cations were analyzed by ion chromatography using a Dionex ICS2000 instrument at a 1 mL/min eluent flow rate. A CG12A pre-column, followed by a CS12A analytical column was used with an eluent of 35 mM methanesulfonic acid. DOC was determined by a TOC-V Total Organic Carbon Analyzer (Shimadzu Scientific Instruments). Inorganic nitrogen species were quantified spectrophotometrically using an AQ2 Discrete Analyzer (Seal Analytical) following the EPA-103-A Rev.10 method for ammonium (LoD 0.004 mg-N/L, range 0.02–2.0 mg-N/L) and EPA-127-A method for NO_3_^–^/NO_2_^–^ (LoD 0.003 mg-N/L, range 0.012–2 mg-N/L). TC and TN in soil pore water was determined with a TOC-V/CSN connected to a TNM-1 unit (Shimadzu Scientific Instruments) following a method based on Standard Methods 5310B and ASTM D8083. Methane δ^13^C was measured with a Thermo Fisher Scientific Precon unit interfaced to a Thermo Fisher Scientific Delta V Plus isotope ratio mass spectrometer (Thermo Fisher Scientific) at the Stable Isotope Facility at U.C. Davis (Yarnes, 2013). DIC concentrations and δ^13^C of DIC measurements were measured on the SHIVA platform in the EcoLab Laboratory (Toulouse, France). The δ^13^C of DIC was analyzed using a mass spectrometer (Isoprime 100, Elementar) coupled with an equilibration system (MultiFlow-Geo, Elementar). Samples were acidified using phosphoric acid and flushed with helium. Standards included Na_2_CO_3_ and NaHCO_3_ as well as internal water standards. All standards were analyzed every 8 samples to check for instrument stability. All samples were analyzed in replicates.

The fractionation factor α was derived with the formula

$$\begin{array}{c} \alpha =\frac{\delta_{C\,O_{2}}+10^{3}}{\delta_{C\,H_{4}}+10^{3}} \end{array}$$

Trace metal (Mo) soil content was determined by inductively coupled plasma mass-spectrometry (ICP-MS) after acid digestion. Briefly, an acid mix (HF+HNO_3_+HCl) was added to soil samples in acid-cleaned Teflon vials. Soil organic matter was oxidized overnight. After another HCl addition, the sample was microwaved and the top liquid was decanted and evaporated on a hotplate. Repeated acid addition and evaporation concentrated the soil metals. A diluted sample was then measured on an iCAP-Q (Thermo Fisher Scientific) with Mo calibrated for 0.018–126 ppb (3% error range).

*E_*h*_ calculations.* The partial pressure of CO_2_ and CH_4_ ($\frac{p_{C\,O\,2}}{p_{C\,H\,4}}$) in soil gas samples was used to derive the equilibrium redox potential (*E*_*h*_) between the redox couple. For each depth interval, *E*_*h*_ was pH-corrected. All calculations were based on the equation CO_2_ + 4 H_2_ = CH_4_ + 2 H_2_O for methanogenesis.

$$\begin{array}{c} E_{p\,H}^{0}=E_{p\,H,0}^{0}-\left(\frac{0.059\,\nu_{H}^{+}}{n}\right)\,p\,H \end{array}$$

The standard potential at pH = 0 ($E_{p\,H,0}^{0}$; Bratsch, 2009), the stoichiometric coefficient ν_*H*_ = 8, and the number of electrons transferred per 1 mole of CO_2_ and CH_4_ (*n* = 8) were used to derive the standard potential at distinct soil depths ($E_{p\,H}^{0}$). From there, we calculated *E*_*h*_ using the Nernst formula. The temperature (*T*) was set to 298 K and we used 8.314 J mol^–1^ K^–1^ for the ideal gas constant (*R*) and 96,485 C mol^–1^ for the Faraday constant (*F*) according to Stumm and Morgan (2012). We used partial pressure as proxy for the activity of the redox species.

$$\begin{array}{c} E_{h}=E_{p\,H}^{0}-2.303\,\left(\frac{R\,T}{n\,F}\right)\,l\,o\,g\,\left(\frac{p_{C\,H_{4}}}{p_{C\,O_{2}}}\right) \end{array}$$

### DNA Extraction, Amplification, and Amplicon Sequencing of 16S rRNA and *mcrA* Genes

Soil DNA was extracted from stored frozen samples using a NucleoSpin Soil DNA extraction kit (Macherey-Nagel GmbH). PCR was performed with the archaeal-bacterial primers 515F/909R (Tamaki et al., 2011) and *mlas/mcrA*-rev (Steinberg and Regan, 2009) to target methanogenic Euryarchaeota. For 16S rRNA gene amplification reactions, we used 0.3 μM of forward and reverse primers, 0.2 mg/L bovine serum albumin, and 1× GoTaq Green Master Mix (Promega). The thermal cycling conditions were the following: Initial denaturation at 95°C for 5 min, 25 cycles of denaturation at 94°C for 30 s, annealing at 52°C for 1 min, and extension at 72°C for 1 min, as well as a subsequent final elongation step at 72°C for 10 min. The reaction chemistry for *mcrA* amplification reactions was used as described elsewhere (Steinberg and Regan, 2009). Thermal cycling included denaturation at 95°C for 3 min, 5 cycles of denaturation at 95°C for 30 s, annealing at 48°C for 45 s, and extension at 72°C for 30 s, accompanied by another 30 cycles of 95°C for 30 s, 55°C for 45 s, 72°C for 30 s, and one final elongation at 72°C for 10 min. Samples were multiplexed (Herbold et al., 2015), normalized (SequalPrep kit, Invitrogen), and sent for sequencing to the DNASU core facility, with 2× 300-bp paired-end Illumina MiSeq (Tempe, AZ).

For archaeal 16S rRNA and *mcrA* gene quantification, the reaction mix consisted of 4 mM MgCl_2_, 1.5× CXR reference dye, 0.3 μM of forward and reverse primer, 0.2 mg/L bovine serum albumin (to reduce inhibition interference) and 1× GoTaq qPCR master mix (Promega). Replicate reactions were run on a QuantStudio 3 real-time PCR system (Applied Biosystems). The primers ARC787f/ARC1059r (Yu et al., 2005) and *mlas/mcrA*-rev (Steinberg and Regan, 2008) were used. Optimal qPCR cycling conditions in reactions with ARC787f/ARC1059r were found to be denaturation at 94°C for 10 min, 45 cycles of 94°C for 10 s and 60°C for 30 s. Cycling stages for *mlas/mcrA*-rev comprised primary denaturation at 95°C for 2 min, followed by 40 cycles of 95°C for 30 s, 55°C for 45 s, 72°C for 30 s, and 83°C for 8 s. Target products amplified by qPCR were confirmed by gel electrophoresis. Standard curves were created with the efficiencies *E* = 1.01–1.03 for *mcrA* and *E* = 0.82–0.88 for archaeal 16S, and good linearity (*r*^2^ > 0.99) in the measurement range of the samples.

### 16S rRNA and *mcrA* Phylogenetic Analyses

Sequences were merged and demultiplexed with an in-house script developed in R. All subsequent analyses were conducted on the Qiime 2 platform^1^ (Caporaso et al., 2010). After dereplication and *de-novo* chimera-filtering, open-reference clustering was applied at a 97% identity level and using the SILVA database (release 128). This reduced the mean and median of sequences per sample from 87,379 and 70,016 to 54,155 and 44,371, respectively. Singletons were removed with the *feature-table filter-features* command. An alpha-rarefaction curve was created for every sample as a visual proxy for species richness (Supplementary Figure 3). Taxonomic classification was done using VSEARCH consensus classifier with SILVA’s 99 majority taxonomy (release 128). Percentage identity was set to 0.94, min-consensus was set to 0.6, and maximum hits accepted were 10. A phylogenetic tree was constructed *de-novo* with the built-in module FastTree 2 (Price et al., 2009). Ammonia-oxidizing microbes were classified based on their affiliation to Thaumarchaeota and *Nitrosomonadaceae*/*Nitrospiraceae* 16S rRNA gene sequences (Supplementary Table 1).

We evaluated methanogen groups based on the alpha subunit gene encoding the MCR enzyme (*mcrA*), which catalyzes the final step in methanogenesis (Ermler et al., 1997). Sequences of the *mcrA* gene were dereplicated using the -*fastx_uniques* command in USEARCH (Edgar, 2010). *Unoise3* was applied to denoise and to filter chimeras. Singleton sequences were retained (-*minsize 1*) to include very low abundance OTUs. The sequences were translated and frameshift-corrected by Framebot (Wang et al., 2013) with a low loss (<10%) of sequences. Subsequent clustering was administered using an 85% identity level (Yang et al., 2014). The -*cluster_fast* algorithm (variant of UCLUST) was fed with centroid sequences, which were derived from a custom-made database. The database was built with sequences from the FunGene repository^2^ in January 2018, and complemented with additional reads from tropical mangroves (Taketani et al., 2010) and a peat swamp forest (Kanokratana et al., 2010). The output of the -*cluster_fast* command was used to make an OTU table, to classify centroid sequences, and to derive core diversity metrics. At this stage, OTUs with 5 or less sequences over all soil layers were removed. To classify the ∼150 amino acids long centroid sequences, we tested two different search algorithms on their performance with a subset of our sample data. The standalone version of BlastP (2.7.1+) and HMMer 3.0 did not show substantial differences in sensitivity and specificity as noted previously (Orellana et al., 2014). Hence, BlastP was run on the non-aligned centroid queries and typically attained *e*-values of 10^–80^ or lower (Takeuchi et al., 2011; Penton et al., 2015). We refined the evaluation of sequence similarity cutoffs for methanogen taxonomic levels (Steinberg and Regan, 2008) by (i) including 102 methanogen species that were all represented by *mcrA* sequences from isolates in swissprot database, (ii) extending the taxonomy to 14 methanogen families, (iii) using the Jone-Taylor-Thornton (JTT) algorithm, and (iv) introducing archaea-adjusted gamma-distribution based on an elongation factor specifically determined for archaea (Gaucher et al., 2001). Distance matrices and phylogenetic trees were derived in MEGA Version 7.0.26. The phylogenetic tree was constructed by aligning sequences while ambiguous positions were removed for each sequence pair. Distances were calculated using the JTT method. Phylogenetic trees of 16S and *mcrA* sequences were overlaid and found to be congruent (Supplementary Figure 4). The overall workflow including the molecular analysis pipeline is visualized in Supplementary Figure 5.

### Microcosm Incubations and Activity Measurements

In a glove box with a reducing, O_2_-free atmosphere (0.5% H_2_ in N_2_), roots and coarse particles (>5 mm) were removed from peat soil, and soil diluted 1:10 in anoxic, sterile 18.2 MΩ × cm water. The slurry was well homogenized, dispensed to culture vials, and sealed with butyl rubber stoppers. Next, an anoxic stock solution of NO_2_^–^ was injected (final concentration 200 μM) to a subset before all vials’ headspace was flushed with pure N_2_. *In-situ* NO_2_^–^ would be supplied steadily from aerobic reactions. But in anoxic microcosms there is no such steady supply and the presence of NO_2_^–^ had to be mimicked, which we did by one single NO_2_^–^ pulse for simplicity. Soil slurries were agitated briefly to disperse the NO_2_^–^ and incubated under dark and static conditions at room temperature for a total of 3 weeks. Headspace (200 μL) was sampled in even time intervals with a gas-tight, N_2_-purged syringe (VICI Precision Sampling) and injected into a gas chromatograph (GC, SRI Instruments) equipped with a flame-ionization detector (FID). Two continuous HayeSep-D columns were kept at 90°C (oven temperature) with N_2_ (UHP grade 99.999%, Praxair Inc.) as carrier gas and H_2_ for FID combustion. Methane concentration measurements were calibrated with customized standard mixtures (Scott Specialty Gases, accuracy ± 5%) over a range of 5–5,000 ppmv. Gas phase concentrations were corrected using Henry’s law and the dimensionless concentration constants *K_*H*_^*cc*^*(CH_4_) = 0.0342 to account for gas dispersed into the aqueous phase at 25°C. Methane production rates were calculated based on the linear region of the concentration curve for manipulated and control incubations.

### Soil Enrichments

Because N_2_O may be derived from NO_2_^–^ via denitrification (see Discussion), we chose the two sites that showed the most contrasting sensitivity to NO_2_^–^ additions (SJO and BVA). From these soils, we derived methanogenic enrichment cultures as previously described (Cadillo-Quiroz et al., 2008) in order to reduce microbial complexity and exposed them to N_2_O by headspace injections. For our tests, culture vials were pressurized with an H_2_/CO_2_ (80/20) mixture (up to 12 PSI) and N_2_O was injected to ∼500 ppm final headspace concentration. Methane and N_2_O changes were monitored by GC injections as described before. For N_2_O detection, an electron-capture detector (ECD) was used in line with the FID. Once N_2_O levels dropped below detectability, the cultures were spiked again with 500 ppm N_2_O. Turbidity (OD_600_ measurements) and hence biomass growth was generally very low for enrichments and microbial growth was therefore confirmed by microscopy. Enrichments were incubated at 30°C.

### Statistical Analyses

All basic statistical tests were performed with JMP Pro software (Version 13.1.0, SAS Institute Inc.). Raw sequence counts were normalized by Johnson transformation. Principal components (Supplementary Figure 6) were explored using the multivariate analysis toolbox on JMP. PERMDISP and PERMANOVA were conducted on the Qiime 2 platform using the *diversity beta-group-significance* command including pairwise testing on 9999 permutations. Alpha and beta diversity indices were calculated using the *diversity core-metrics-phylogenetic* command configured with sampling depths according to individual sample sets. *McrA* phylogeny was visualized with Krona (Ondov et al., 2011). Spearman’s rank multivariate analysis (Supplementary Table 2) was conducted on *mcrA* sequence reads using JMP. Plotting and regression analysis was done with the MATLAB R2018a software (Version 9.4.0.813654, Mathworks Inc.).

## Results

### PMFB Peatlands Show Differing Soil CH_4_ Profiles, DOC Loads, and Inorganic Nitrogen Levels

**FIGURE 1:**
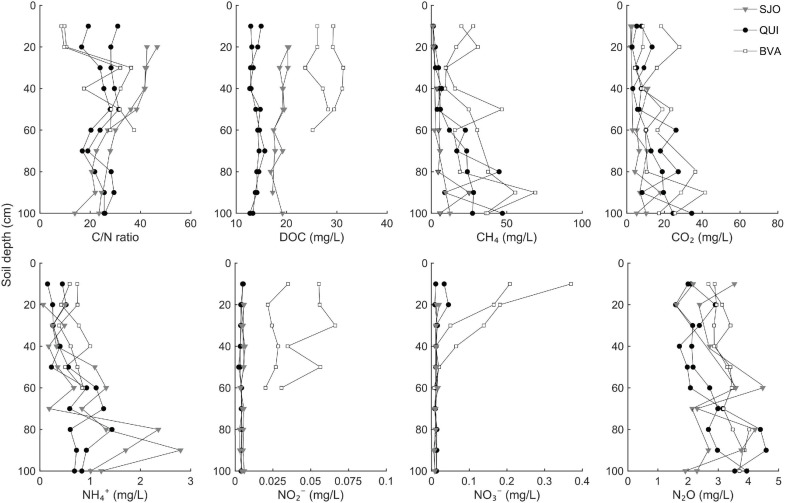
Levels of carbon and nitrogen along soil profiles of three tropical peatlands (QUI, SJO, BVA). Two separately sampled (duplicate) profiles from Quistococha (QUI), San Jorge (SJO), and Buena Vista (BVA) were each set up to sample pore water and soil gas. Ions, total carbon and nitrogen abundances, and DOC were determined from pore water. Gas concentrations were measured in equilibrated gas samples. Liquid samples in BVA were not collected below 60 cm due to blocked sampling paths by a dense network of roots.

Equilibrated soil gas analyses (see section “Materials and Methods”) showed that CH_4_ maxima per site were up to 25 g/L in SJ, 47 g/L in QUI and 69 g/L in BVA, predominantly in deep peat (Figure 1). All sites contained roughly 10 times higher N_2_O levels than the mean ambient atmosphere (∼320 ppb, Stocker et al., 2013). We observed that particularly soil columns of the less acidic site (BVA) showed increasing N_2_O concentrations with soil depth (Figure 1). Tests for pore water NO_2_^–^ concentrations in SJO and QUI did not show statistically significant differences from the method’s limit of detection (LoD, 0.01 mg/L), and only BVA samples yielded NO_2_^–^ levels above the LoD (*p* < 0.05). Nitrate reached highest concentrations in shallow soil layers, with maxima at 0.4 mg/L (BVA), 0.05 mg/L (QUI), and 0.02 mg/L (SJO), but N_2_O was generally the dominant nitrogen oxide. Ammonium (NH_4_^+^) showed a vertical pattern opposite to NO_2_^–^ and NO_3_^–^ (Figure 1). Concentrations increased by roughly a factor of 4 from top to bottom layers in SJO, and followed a similar, but less pronounced, trend at the other two locations. Since molybdenum (Mo) is an essential trace metal in N_2_ fixation (Mo cofactors are the active site in nitrogenases), we determined Mo abundances in top soils (0–30 cm depth) to exclude the possibility of Mo limitation. Acid-digested peat extracts yielded Mo concentrations of 0.53 ± 0.01 ppm (QUI), 1.25 ± 0.2 ppm (SJO), and 1.32 ± 0.1 ppm (BVA, all *n* = 3). Dissolved organic carbon (DOC) profiles derived from soil pore water differed significantly across sites (ANOVA, *p* < 0.05, Figure 1). Highest DOC concentrations were found in BVA peat (up to 29.1 mg/L). Unlike DOC profiles, C/N ratios resulted in significant variation along soil depth. A trend of increasing C/N values with increasing depth was observed in BVA where the ratio at the 60 cm-layer was ∼3.6 times that of surface layers (Figure 1). In contrast, the C/N profile at SJO decreased with increasing depth with values from uppermost and lowermost layer being ∼2.4-fold difference.

### Overall Microbial Community Composition Along Peat Soil Profiles

**FIGURE 2:**
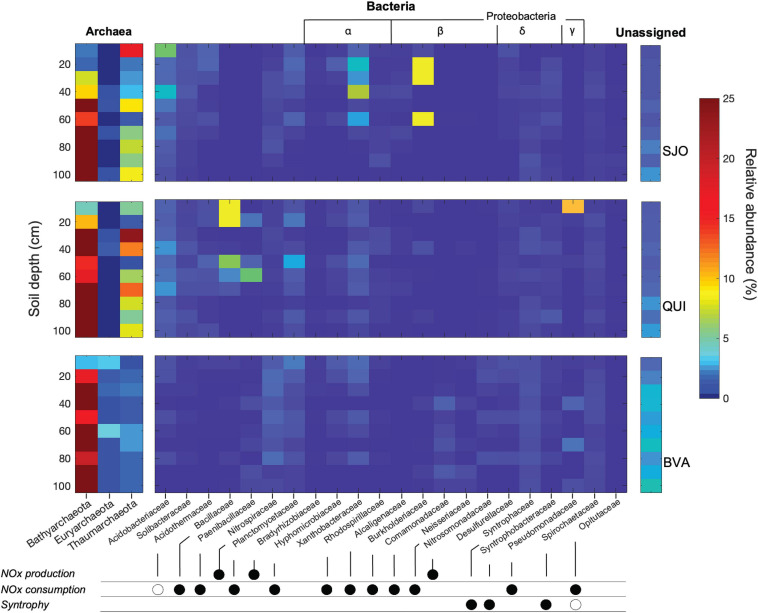
16S rRNA gene relative abundance heatmap of archaeal (left) and bacterial (right) taxa with known nitrogen metabolic potential. Archaeal 16S rRNA gene relative abundances were summarized on the phylum level with Bathyarchaeota, Euryarchaeota, and Thaumarchaeota consistently making up >90% of all archaeal taxa. Only bacterial taxa at the family level that were either most dominant (at least 2% of total OTUs), had the potential of a syntrophic relationship to methanogens, or were affiliated to known metabolic reactions involving NO_*x*_ are depicted. Confirmed metabolic capacities to produce NO_*x*_, consume NO_*x*_, or for a syntrophic lifestyle are marked with closed circles. Hypothesized metabolic capacities (unsupported by pure cultures) are marked with open circles. The number of unassigned sequences (no match in the SILVA database) increased notably in deeper layers. Relative abundances for individual cores and bacterial metabolic capacities at the family level are provided in Supplementary Table 1.

The geochemical diversity of the three peatlands was reflected in their contrasting microbial community composition. A total of 4,980,584 sequencing reads were clustered to 16S rRNA operational taxonomic units (OTUs) with 3% identity radius for taxa assignment on the genus level or above. SJO with 6,636 OTUs had the lowest richness while BVA yielded about three times as many OTUs (18,649). We focused henceforth on microbial groups putatively relevant to the cycling and vertical distribution of soil NO_*x*_ (Supplementary Table 1) or that were highly abundant (Figure 2). We assumed ecological coherence within the family taxonomic rank as proposed elsewhere (Philippot et al., 2010) but emphasize that likely not all taxa of a Family share NO_*x*_-cycling traits. Bathyarchaeota was a very abundant phylum frequently making up 20% of total OTU counts, predominantly in deeper regions of the peat soils. In contrast, Euryarchaeota were less abundant and did not show a depth preference. The surface soil layer in BVA cores was among the richest in Euryarchaeota with ∼3% of total OTUs. In QUI and SJO, maxima in Euryarchaeota OTUs (1.5 and 1%, respectively) were found in moderate depths (Figure 2). *Xanthobacteraceae* relative abundances peaked at mid profile depth in QUI (5.4 and 3.5% of total OTUs in each core). In SJO peat soil, the group reached even higher values of 17.9% and remained at ∼1% of total OTUs in a depth of 75 cm before ceasing in the bottom layers. BVA showed maximum relative abundances for *Xanthobacteriaceae* (4.7 and 5.4%) exclusively in shallow layers. As potential nitrifiers (NO_*x*_ producing), Thaumarchaeota showed high relative abundances in QUI and SJO (up to 23.7 and 16.8%), and low relative abundances in BVA. *Nitrospiraceae* were most abundant in BVA in soil layers (3.3–3.7%) slightly below the top layer (15–25 cm in both duplicate cores). Potentially denitrifying (NO_x_^–^ consuming) *Bacillaceae* and *Paenibacillaceae* seem to be a unique occurrence in the palm swamp of QUI with relative abundances of up to 8.4 and 2.7%, respectively.

### Methane Isotopic Signatures and Soil Redox Potential

**FIGURE 3:**
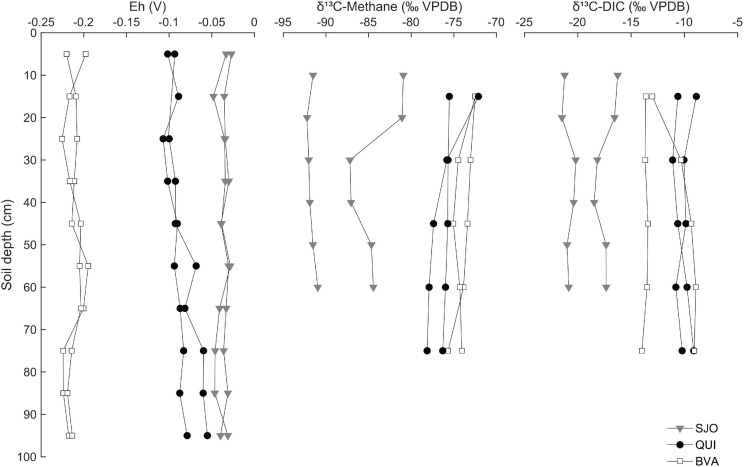
Redox gradient and carbon isotope composition of CH_4_ and dissolved inorganic carbon along soil profiles of three tropical peatlands (BVA, QUI, SJO). Redox potential (E_*h*_) is based on the ratio of the CH_4_-CO_2_ redox couple and soil pH-corrected. VPDB, Vienna Pee Dee Belemnite. Each data point corresponds to one replicate soil core.

The isotopic composition (δ^13^C) of dissolved CH_4_ and inorganic carbon (DIC) shows active methanogenesis in all peatlands and points to signatures of distinct pathways. Values of δ^1^^3^C in CH_4_ and DIC from BVA and QUI were indistinguishable from each other but distinct from SJO, which had a more depleted δ^13^C signature (Figure 3). In SJO, δ^1^^3^C values in CH_4_ also showed variation between duplicate cores with a core showing a sudden depletion at 20 cm depth by 6‰ (Figure 3).

The average redox potential (*E*_*h*_) based on the CH_4_/CO_2_ pair decreased with more positive values in SJO, followed in order by QUI and BVA (Figure 3), consistent with observed levels of CH_4_ emissions and abundance trends of the *mcrA*/16S rRNA fraction (see below). Along soil profiles, *E*_*h*_ in SJO decreased from –0.03 to –0.035 V, increased in QUI from –0.098 V to –0.067 V, while BVA showed negligible change at –0.21 V.

### Methanogen Diversity and Distribution

**FIGURE 4:**
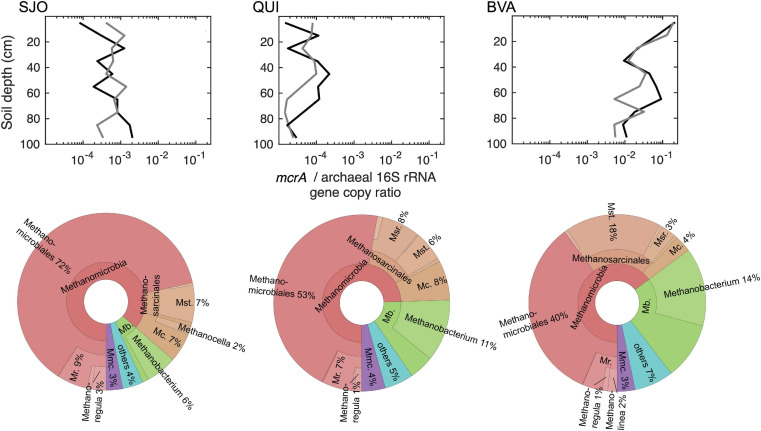
*McrA*/archaeal 16S rRNA gene copy ratios along soil depth (left) and *mcrA* phylogeneticphylogenetic distribution of cumulative reads at all levels (right) in each peatland. Gene copy ratios are based on qPCR analysis and data from both soil cores are shown (dark and light gray lines). The gene ratios were used to better illustrate methanogen proportions relative to the total number of archaea, which explicitly revealed differences between peatlands. Absolute gene quantities ranged from 1.3 × 10^4^ (SJO) to 1.8 × 10^9^ (BVA) *mcrA* gene copies per g of dry soil and 8.7 × 10^5^ (SJO) to 7.3 × 10^11^ (QUI) archaeal 16S rRNA gene copies per g of dry soil. Relative abundance percentages refer to the total number of OTUs recovered at each site. Higher level taxa were collapsed if these were comprised of 100% the lower-level taxa. Mr., *Methanoregulaceae*; Mst., *Methanosaetaceae*; Msr., *Methanosarcinaceae; Mc., Methanocellaceae; Mb., Methanobacteriaceae;* Mmc., *Methanomassiliicoccaceae*.

The methanogen communities in BVA and SJO diverged the most and BVA exhibited the highest methanogen diversity of all studied peatlands as evaluated through the *mcrA* gene (Figure 4). PERMANOVA tests on *mcrA* amplicons from all cores indicated significant differences among peatlands. Site replicates were not distinguishable (*F* = 1.11, *p* = 0.2494), but values across sites showed increasing dissimilarity with BVA closer but distinct to QUI (*F* = 7.5 ± 1.6), *p* < 0.0005), and further from and more distinct to SJO (*F* = 11.4 ± 0.7, *p* < 0.0001). *McrA* amplicon analysis identified 264, 509, and 1,104 OTUs from SJO, QUI, and BVA, respectively. Alpha diversity analysis confirmed BVA as the site with the most diverse methanogen community (Shannon index, SI = 4.3 ± 0.4) followed by QUI (SI = 3.6 ± 0.4), and SJO (SI = 2.7 ± 0.5) as the least diverse. This order reflects the more favorable physiological conditions for methanogenesis under low *E*_*h*_ (Figure 3).

Figure 4 illustrates Methanomicrobiales as most dominant across sites (40–72%) particularly in SJO, while Methanobacteriales reached its highest fraction (25%) in BVA but lowest (7%) in SJO. Furthermore, Methanomicrobiales sequence alignment on the amino acid level showed their clustering with environmental clones outside the established phylogeny (Supplementary Figure 7), with OTUs less than 67% similar to any known family and likely novel groups in the order. Additionally, OTU fractions (Figure 4) also evidenced the putative acetoclastic contribution by *Methanosaetacea* and *Methanosarcinacea* particularly in BVA (∼21%), a lesser degree in QUI (14%) and SJO (8%). In BVA, the site with highest pH and minerotrophic conditions, *Methanosaetaceae* (including *Methanothrix*) represented nearly a fifth (18%) of the community. Meanwhile, in QUI *Methanosarcinacea* (including *Methanosarcina*) made up a bigger fraction (8%) than *Methanosaetacea* (6%), suggesting differential acetoclastic contributions likely related to acetate availability and the significantly higher acetate affinity of *Methanothrix* over *Methanosarcina* (Min and Zinder, 1989; Jetten, 1992). Quantitative PCR (qPCR) gene copy results exhibited distinct abundances and vertical distributions across sites. All three peatlands showed distinct ranges in *mcrA* copies, orders of magnitude apart (Figure 4) with the following order: BVA > SJO > QUI. Moreover, the *mcrA* relative frequency decreased notably in BVA by 2 orders of magnitude from the surface to 35 cm depth.

### Composition of Methanogenic Community Along Vertical Profiles Correlates With NO_*x*_ Values

**FIGURE 5:**
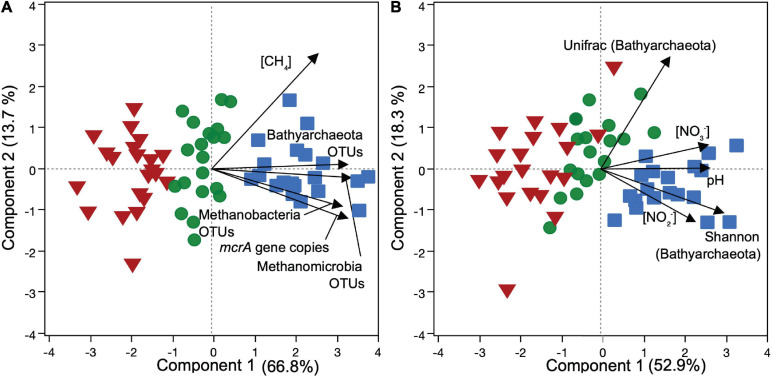
PCA ordination plots of microbial and environmental data from three soil profiles of contrasting Amazon peatlands. Samples were derived from QUI (red triangles), SJO (green circles) and BVA (blue squares) with abbreviations as in Figure 1. Methane concentration ([CH_4_]), 16S rRNA gene OTU relative abundances, and *mcrA* gene copy number **(A)**, and Bathyarchaeota alpha (Shannon) and beta (Unifrac) diversity indices, NO_*x*_ concentrations ([NO_2_^–^], [NO_3_^–^]) and pH **(B)** were evaluated against microbial distribution. We used Bathyarchaeota abundance and diversity indices as a not yet physiologically resolved close archaeal group important to evaluate but not as assumed methanogens.

Our statistical analyses exhibited different correlation trends between NO_*x*_ and the relative abundance of distinct methanogenic groups along soil profiles. Together with the principal components analysis (PCA, Figure 5), Spearman correlation pointed to an overall positive correlation of NO_3_^–^ with methanogen relative abundances, while NO_2_^–^ did not show significant relationships. Specifically, PERMANOVA tests indicated a significant shift of the overall *mcrA*-sequenced methanogenic community between the depth interval that was relatively enriched in NO_*x*_ (0–50 cm) and the one relatively depleted in NO_*x*_ (50–100 cm) for both cores in BVA (*F* = 5.39, *p* = 0.0170), QUI (*F* = 4.13, *p* = 0.0190), and SJO (*F* = 3.71, *p* = 0.0493). The differences in variation were unaffected by dispersion (*p* > 0.05). Spearman’s rank further indicated sequence counts of *Methanosaetaceae* and *Methanosarcinaceae* correlated positively with NO_3_^–^ (*ρ* = 0.76 and 0.72 correspondingly, *p* < 0.005) in SJO, while *Methanoregulaceae* and NO_2_^–^ correlated negatively (*ρ* = −0.79, *p* < 0.005) in BVA (Supplementary Table 2 and Supplementary Figure 8). Other geochemical parameters, such as pH (|ρ| < 0.49), phosphate (|ρ| < 0.56), sulfate (|ρ| < 0.50), and ammonium (|ρ| < 0.47), did not reveal more significant relationships with any of the selected methanogen taxa (Supplementary Table 2). Principal component analysis showed well separated data points clustered into the three peat soils (Figure 5). OTU relative abundances of Bathyarchaeota, Methanomicrobia, and Methanobacteria were similarly associated to principal component 1 (PC 1), while CH_4_ concentration was related to PC 2.

Given the negative correlation of NO_2_^–^ and a methanogenic group in BVA and the overall variation of methanogenic communities in NO_*x*_ gradients across all sites, we conducted a further in-depth evaluation beyond field measurements testing methanogen sensitivity to NO_*x*_ species.

### CH_4_ Production Potentials in SJO, QUI, BVA Peat Soils Show Different Responses to NO_2_^–^ Amendment and N_2_O Halts Methanogenesis

**FIGURE 6:**
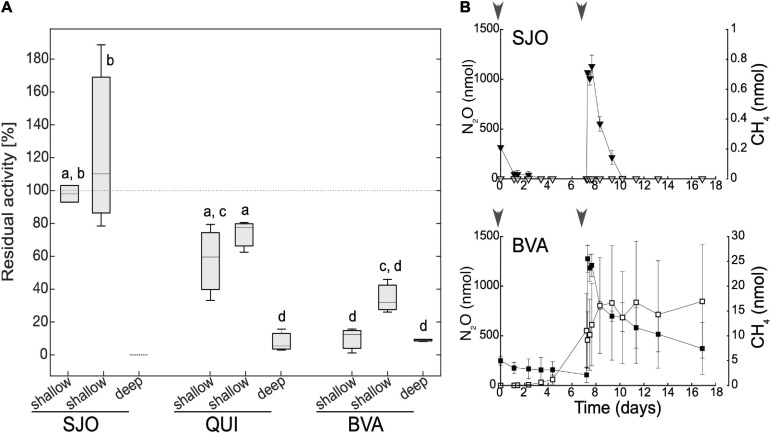
Effect of NO_2_^–^ amendment on methanogenic activity in peat soil incubated under anoxic conditions **(A)** and effect of N_2_O on enrichment cultures **(B)**. **(A)** For each site, two shallow (0–15 cm) and one deep (15–30 cm) soil slurry samples were incubated in triplicates. Residual activity denotes fractional CH_4_ production relative to controls without additions. Dotted line for SJO’s deep soil denotes CH_4_ levels below detection limit. Levels not reporting the same letter are significantly different (Student’s *t*-test, *p* = 0.05). **(B)** Nitrous oxide (full symbols) and CH_4_ (open symbols) dynamics in enrichment cultures from SJO and BVA. Units are nmol per incubation vessel. Methane concentrations in SJO remained below detection throughout the illustrated incubation period. Gray arrowheads denote N_2_O addition time. Error bars represent one standard deviation.

Our tests on the effects of NO_2_^–^ on methanogenesis rates in amended (200 μM NO_2_^–^) anoxic soil slurries or effects of N_2_O on methanogenic enrichment cultures revealed responses consistent with our field observations showing inhibitory effects and variable responses. We found that NO_2_^–^ exerted differential, mostly negative effects on the CH_4_ production potential across the contrasting tropical peatlands (Figure 6A). Samples from deeper soils showed the highest residual activity reduction (down to ∼10% or below detection) across all sites. However, shallow, presumably most active, soil samples showed the most variation in CH_4_ output. The oligotrophic site (SJO) with the lowest potential methanogenic rates was not affected or instead stimulated marginally (90% or >100% residual activity, respectively). The intermediate site (QUI) had a mild or moderate reduction (retaining ∼60–80% residual activity), and the minerotrophic and most CH_4_-producing site (BVA) had the harshest reduction (∼15–30% residual activity). The latter is consistent with our expectations based on the relatively steeper field NO_2_^–^ gradients observed in BVA.

Using recently developed methanogenic enrichments from BVA and SJO, we found N_2_O inhibits methanogenesis, with a strong threshold-based behavior (Figure 6B). Under H_2_/CO_2_ overpressure (controls, no organic substrates added) and without added N_2_O, SJO, and BVA enrichments showed linear CH_4_ production by 0.6 and 4.7 nmol day^–1^, respectively. Enrichments receiving N_2_O injections showed N_2_O reduction concomitant to CH_4_ production. Methane accumulation did not progress in a linear fashion as seen in controls and halted when cultures were spiked with 28 μM N_2_O (aqueous concentration). Methane formation appeared to resume when N_2_O fell below a threshold of ∼10 μM in BVA (after 7 days) and to < 0.004 μM in SJO (after 17 days). The recovery of methanogenesis was less pronounced in SJO whose enrichment had low methanogenic biomass likely leading to lagging periods longer than the 7 days between N_2_O injections used in our assays (Figure 6B). N_2_O consumption observed in the SJO enrichment (pH 3.5) is noteworthy because it occurred despite the known ceasing of N_2_O reductase activity at pH < 5 (Liu et al., 2010, 2014). These results show that N_2_O—a stable nitrogen oxide species under peat soil conditions (contrary to NO_2_^–^)—that can only be removed enzymatically, inhibits methanogenesis if present above a distinct threshold. We determined thresholds of 0.004–10 μM pointing at a wide range in individual N_2_O sensitivity among methanogens.

## Discussion

### Contrasting Overall CH_4_ Patterns Across SJO, QUI, and BVA Peatland

Our study revealed distinct vertical patterns in the distribution of several carbon (including methane) and nitrogen pools among contrasting Amazon peatlands. In the acidic nutrient-poor SJO peats, low NO_3_^–^ concentrations throughout the soil profile raised the C/N ratio, indicating relative nitrogen scarcity (Hall and Matson, 2003). SJO peatland also yielded the most oxidized CH_4_/CO_2_ redox balance and showed signs of methane oxidation in the isotopic abundances. The “hook” shape of the ^13^C-CH_4_ profile (Figure 3) likely indicates aerobic methane oxidation, which preferentially converts ^12^CH_4_ to CO_2_, resulting in an enrichment of ^13^CH_4_ in the residual dissolved CH_4_ pool. A similar concentration pattern as in oligotrophic SJO has been also described in an oligotrophic tropical peatland in Panama (Holmes et al., 2015). The depletion of CH_4_ pools could explain why moderate *mcrA* gene frequencies would not lead to higher CH_4_ soil gas concentrations in SJO peats. In the mildly acidic QUI peatland, the observed C/N ratios are consistent with previous measurements underlining the moderate to poor trophic conditions relative to the other peat soils (Lawson et al., 2014). The steep decrease of CH_4_ concentrations in surface levels but absence of a strong signature of CH_4_ oxidation in the isotopic data suggest that CH_4_ is lost from soils perhaps mainly through diffusion from water saturated soil to the atmosphere or through palms and trees as reported for this site (Van Haren and Cadillo-Quiroz, 2016). Aerobic oxidation of methane likely operates in QUI as methanotrophic sequences were detected in this and previous study (Finn et al., 2020), however, frequent near or above soil surface water table in this site likely limit this activity to dryer seasons or elevated soils. The minerotrophic, near-circumneutral pH, peat soils of BVA were characterized by relatively high NO_3_^–^ concentrations in shallow soil layers, leading to lower C/N ratios and a higher nitrogen nutrient richness that in the other sites. This trophic condition could be the basis of the greater diversity and relative abundance of methanogens observed in BVA soils, which also presented the most favorable redox conditions for methanogens. As a result, relatively high CH_4_ concentrations up to more shallow soil layers lead to increased emission potential. Aerobic methane oxidation is likely similarly limited by water table and diffusion processes as in QUI, but more research is needed on that component. The isotopic CH_4_ and DIC proportions yield fractionation factors (α) of 1.075 ± 0.0015 in SJO, 1.071 ± 0.0009 in QUI, and 1.067 ± 0.0015 in BVA (*SD*, *n* = 10–12), and are within a previously reported range (Holmes et al., 2015). According to the current consensus, α < 1.055 and α > 1.065 are characteristic for environments dominated by acetate-using (acetoclastic) or H_2_-using (hydrogenotrophic) methanogenesis, respectively (Whiticar, 1999). Thus, the source of CH_4_ is preferentially H_2_/CO_2_ in the studied peatlands, with a more noticeable contribution of acetoclastic methanogenesis in BVA.

### Source Pathways and Microbial Origin of NO_*x*_ in Peat Soils

The dynamics of the nitrogen cycle in tropical peatlands has not been detailed but our measurements allow us to postulate some likely pathways leading to NO_*x*_ production by different microbial groups. Ammonium (NH_4_^+^) sources could include release from organic matter through ammonification, N_2_ fixation, or DNRA. The latter would not serve as a net source of dissolved inorganic nitrogen, because NH_4_^+^ is produced from NO_3_^–^. Plant debris with low C/N ratio tends to trigger net N mineralization (Manzoni et al., 2010) which would result in increasing NH_4_^+^ concentrations as observed in SJO deep peat. Also, microbial fixation of N_2_ mediated by the Mo-based nitrogenase enzyme is another likely source of NH_4_^+^. The unusual temperature dependence of the enzyme grants a high N_2_-fixing capacity to tropical peats (Houlton et al., 2008), which can lead to accumulatio of inorganic N. In fact, the measured Mo content among sites is above the limiting range for N_2_ fixation by free-living heterotrophic bacteria in tropical forest soils (Barron et al., 2008).

In contrast to NH_4_^+^, NO_*x*_ species were more abundant in shallow than deeper soils, irrespective of the site-specific geochemistry, which is consistent with previous reports (Hu et al., 2014; Palmer and Horn, 2015; Tang et al., 2018b). We found that the relative fraction of Thaumarchaeota and *Nitrospiraceae* were highly abundant (Figure 2) and likely play an important role in regulating pulses of NO_*x*_ through peat soils. Thaumarchaeota are mostly ammonia-oxidizing archaea (AOA), while *Nitrosomonadaceae* (genus *Nitrosomonas*) and *Nitrospiraceae* (genus *Nitrospira*) are members of the ammonia-oxidizing bacteria (AOB). AOA and AOB generally prefer inorganic nitrogen sources, even though AOA have been shown to metabolize organic nitrogen, too (Weber et al., 2015). Both AOA and AOB aerobically convert NH_4_^+^ into NO_2_^–^. Several studies have pointed out that AOA are more dominant in acidic soils than their bacterial counterparts (Zhang et al., 2012), which is likely due to indirect pH effects on the preferred substrates (Höfferle et al., 2010; Yao et al., 2011; Daebeler et al., 2014; Weber et al., 2015). The partitioning of both groups across the studied peatlands reflects this pattern with the dominance of AOB over AOA in BVA, and the opposite pattern in mildly acidic QUI and highly acidic SJO. We propose nitrification to be mostly carried out by archaea residing in top (0–30 cm) soil layers of the oligotrophic and acidic sites and AOB-mediated nitrification to prevail in the minerotrophic, closer to neutral pH site. This preferential niche selection would have further implications on the NO_3_^–^ to NO_2_^–^ ratio. Some species of the *Nitrospiraceae* (AOB) are capable of oxidizing NO_2_^–^ further to NO_3_^–^. Therefore, the capacity for complete nitrification (Daims et al., 2015; van Kessel et al., 2015) could explain the higher levels of NO_3_^–^ detected in the NO_*x*_ profile from BVA (Figure 1).

### NO_*x*_ Affect Peat Methanogenesis Directly or Indirectly, via Reaction to N_2_O

Based on the correlation of distinct methanogenic taxa with NO_*x*_ distribution and sensitivity of methane formation to NO_*x*_, we infer an inhibition of microbial methanogenesis and propose 3 plausible explanations. First, the introduction of NO_2_^–^ can change redox potential and thus decrease CH_4_ production. NO_*x*_ have naturally high redox potentials. Methanogenesis operates preferentially at negative potentials and soil communities have been shown to be sensitive to changes in redox conditions (Fetzer and Conrad, 1993; Jugsujinda et al., 2008; Hirano et al., 2013). Moreover, the magnitude of observed inhibition is consistent with the redox gradients measured *in-situ*. Methanogens of BVA soil would experience the greatest difference in redox because the CH_4_/CO_2_ balance in BVA is most reduced (Figure 3), and hence, exposure to oxidized radicals would result in harsh disruption of the redox balance. Second, denitrifying microorganisms can deplete H_2_ or acetate, thus, competing with methanogens for the common substrates. The predominance of the hydrogenotrophic or acetoclastic methanogenic pathway together with the substrate preference of the indigenous denitrifying community (NO_*x*_ consumers, Figure 2) could lead to the apparent pattern (Klüber and Conrad, 1998a; Conrad, 1999). Third, NO_2_^–^ negatively affects methanogens on a physiological level. Here, derivatives of transformation processes from NO_2_^–^ might be stronger agents than NO_2_^–^ itself. Under natural soil conditions, NO_2_^–^ can biotically or abiotically be transformed into NO and N_2_O (Buessecker et al., 2019). Elevated NO concentrations affect a variety of cellular processes, often leading to cytotoxicity (Saraiva et al., 2004). Similarly, N_2_O at ∼0.1 mM levels has been found to be an inhibitor of methanogenesis in environmental samples (Balderston and Payne, 1976). N_2_O has been shown to bind and inactivate vitamin B_12_ (Drummond and Matthews, 1994), a key metabolite for methanogens because its cobalt(I) center serves as a methyl acceptor during methanol to methane transformation (Matthews, 2001). The resistance of some methanogenic groups to N_2_O exposure by vitamin B_12_ synthesis or the presence of complementing phyla that can make vitamin B_12_ (e.g., *Thaumarchaeota*, Doxey et al., 2014) may result in the differential suppression of CH_4_ production across sites.

Conversely, the measured CH_4_ pools may also be indirectly affected by NO_*x*_. For instance, NO_2_^–^-based anaerobic methane oxidation (AOM) could affect the results without direct effects on methanogenesis since our experiment only accounted for net CH_4_ accumulation. However, considering the slow CH_4_ oxidation rates observed in other freshwater experiments (Zhu et al., 2012; Segarra et al., 2015; Valenzuela et al., 2017), it is unlikely that CH_4_ levels can be significantly affected by AOM during our incubations.

Steady-state N_2_O could have retarding effects on CH_4_ accumulation at SJO peatland but is insufficient to affect methanogenesis at BVA. The equilibrated N_2_O soil gas concentrations we measured *in-situ* (Figure 1) correspond to 0.05–0.1 μM N_2_O dissolved in soil pore water. The determined thresholds of ∼10 μM in BVA and approximate <0.004 μM in SJO are above and below the N_2_O soil gas concentrations. This may contribute to the general emission pattern of higher CH_4_ emissions from BVA peat and low CH_4_ emissions from SJO peat (Finn et al., 2020). We should note that the enrichments used for the incubations represented only a fraction of the microbial community present and active *in-situ*. Nevertheless, the NO_2_^–^ or N_2_O effects on CH_4_ accumulation in soil incubations or culture enrichments suggest that complex links between the nitrogen and carbon cycle occur in Amazon peatlands. These may likely comprise interactions of nitrifying microbes and methanogens, which would contribute to the high spatial variation in CH_4_ production observed in peatlands.

## Conclusion

Taken together, the PMFB harbors peatlands with diverse edaphic chemistries, which is especially reflected in pH, DOC, NO_3_^–^ pore water concentrations and C/N ratios of shallow soils. Based on the physicochemical conditions in the waterlogged peats (redox, trace metal availability, substrates) the tropical soils provide feasible conditions for microbial methanogenesis, predominantly via the hydrogenotrophic pathway. The overall microbial communities show strong patterns of vertical stratification in the top 1 m. Thaumarchaeota and *Nitrospira* have similar ecological niches as producers of NO_*x*_ species. Thaumarchaeota are more abundant in the low-pH sites SJO and QUI, whereas *Nitrospira* appear more dominant in the more neutral-pH soil BVA. Our data reveal high potential of novel and diverse methanogens affiliated with the Methanomicrobiales order. We present evidence that soil NO_*x*_ species impact methanogen relative abundances and activities and our laboratory incubations show significant effects of NO_2_^–^ and N_2_O on CH_4_ production.

Our findings may have broad implications on how pulses of nitrogen oxides, which are controlled by nitrifying/denitrifying groups and abiotic factors, could contribute to the spatial and seasonal variation observed in CH_4_ emissions from peatlands of the Amazon. For instance, drought-induced drainage of peatlands may fuel nitrification and the production of nitrogen oxides, which, in turn, lower CH_4_ production. Lowering of the water table and the oxic-anoxic interface would shift the NO_*x*_ profile down toward soil layers with higher methane content. Subsequentially, nitrogen oxides become a more dominant electron acceptor than CO_2_ resulting in increased CO_2_ production and decreased CH_4_ production. With a higher diffusion range than O_2_, nitrogen oxides reach more methanogenic cells and may inhibit them before they actually encounter O_2_. Because of the variable effects of NO_2_^–^ and N_2_O, and the divergent vertical distribution of these compounds, further work is needed to assess effects of NO_*x*_ species on individual soils. Regardless, our study highlights an underestimated link between the aerobic branch of the nitrogen cycle and the anaerobic branch of the carbon cycle yielding a generally negative feedback on methane formation in tropical peat soils. Because Amazonian peatlands represent large stocks of organic carbon, the magnitude of this feedback may be critical to regulate how much carbon in the form of CH_4_ would be exported into the atmosphere.

## Data Availability Statement

All geochemical data can be found in the paper and Supplementary Material. The sequence data have been deposited in the GenBank, EMBL and DDBJ databases as SRA Bioproject PRJEB36841.

## Author Contributions

SB and HC-Q designed the study and wrote the manuscript. SB, JvH, JU, AMH, and HC-Q conducted the field work. SB and AFS performed the molecular work at the laboratory. SB and DRF analyzed the sequence data. SB and ZZ conducted incubations and geochemical analytical work. SB, AMH, and HC-Q analyzed the geochemical data. All authors contributed to the final draft of the manuscript.

## Conflict of Interest

The authors declare that the research was conducted in the absence of any commercial or financial relationships that could be construed as a potential conflict of interest.

## Footnotes

1 https://qiime2.org, releases 2017.12 and 2018.8.

2 http://fungene.cme.msu.edu

## References

[B1] Bai R. Wang J.-T. Deng Y. He J.-Z. Feng K. Zhang L.-M. (2017). Microbial community and functional structure significantly varied among distinct types of paddy soils but responded differently along gradients of soil depth layers. *Front. Microbiol.* 8:945. 10.3389/fmicb.2017.00945 28611747 PMC5447084

[B2] Balderston W. L. Payne W. J. (1976). Inhibition of methanogenesis in salt marsh sediments and whole-cell suspensions of methanogenic bacteria by nitrogen oxides. *Appl. Environ. Microbiol.* 32 264–269. 970945 10.1128/aem.32.2.264-269.1976 PMC170046

[B3] Barron A. R. Wurzburger N. Bellenger J. P. Wright S. J. Kraepiel A. M. L. Hedin L. O. (2008). Molybdenum limitation of asymbiotic nitrogen fixation in tropical forest soils. *Nat. Geosci.* 2 42–45. 10.1038/ngeo366

[B4] Blodau C. (2002). Carbon cycling in peatlands – A review of processes and controls. *Environ. Rev.* 10 111–134. 10.1139/a02-004 33356898

[B5] Bloom A. A. Palmer P. I. Fraser A. Reay D. S. Frankenberg C. (2010). Large-scale controls of methanogenesis inferred from methane and gravity spaceborne data. *Science* 327 322–325. 10.1126/science.1175176 20075250

[B6] Bratsch S. G. (2009). Standard electrode potentials and temperature coefficients in water at 298.15 K. *J. Phys. Chem. Ref. Data* 18 1–21. 10.1063/1.555839

[B7] Buessecker S. Tylor K. Nye J. Holbert K. E. Urquiza-Muñoz J. D. Glass J. B. (2019). Effects of sterilization techniques on chemodenitrification and N_2_O production in tropical peat soil microcosms. *Biogeosciences* 16 4601–4612. 10.5194/bg-16-4601-2019

[B8] Cadillo-Quiroz H. Bräuer S. Yashiro E. Sun C. Yavitt J. Zinder S. (2006). Vertical profiles of methanogenesis and methanogens in two contrasting acidic peatlands in central New York State. *USA. Environ. Microbiol.* 8 1428–1440. 10.1111/j.1462-2920.2006.01036.x 16872405

[B9] Cadillo-Quiroz H. Yashiro E. Yavitt J. B. Zinder S. H. (2008). Characterization of the archaeal community in a minerotrophic fen and terminal restriction fragment length polymorphism-directed isolation of a novel hydrogenotrophic methanogen. *Appl. Environ. Microbiol.* 74 2059–2068. 10.1128/aem.02222-07 18281434 PMC2292581

[B10] Cape J. N. Kirika A. Rowland A. P. Wilson D. R. Jickells T. D. Cornell S. (2001). Organic nitrogen in precipitation: real problem or sampling artefact? *Sci. World J.* 1 230–237. 10.1100/tsw.2001.278 12805792 PMC6084209

[B11] Caporaso J. G. Kuczynski J. Stombaugh J. Bittinger K. Bushman F. D. Costello E. K. (2010). QIIME allows analysis of high-throughput community sequencing data. *Nat. Methods* 7 335–336. 10.1038/nmeth0510-335 20383131 PMC3156573

[B12] Chen J. Hanke A. Tegetmeyer H. E. Kattelmann I. Sharma R. Hamann E. (2017). Impacts of chemical gradients on microbial community structure. *ISME J.* 11 920–931. 10.1038/ismej.2016.175 28094795 PMC5363838

[B13] Cleemput O. V. Samater A. H. (1996). Nitrite in soils: accumulation and role in the formation of gaseous N compounds. *Fert. Res.* 45 81–89.

[B14] Conrad R. (1999). Contribution of hydrogen to methane production and control of hydrogen concentrations in methanogenic soils and sediments. *FEMS Microbiol. Ecol.* 28 193–202.

[B15] Conrad R. (2020). Methane production in soil environments-anaerobic biogeochemistry and microbial life between flooding and desiccation. *Microorganisms* 8:881. 10.3390/microorganisms8060881 32545191 PMC7357154

[B16] Daebeler A. Bodelier P. L. Yan Z. Hefting M. M. Jia Z. Laanbroek H. J. (2014). Interactions between Thaumarchaea, Nitrospira and methanotrophs modulate autotrophic nitrification in volcanic grassland soil. *ISME J.* 8 2397–2410. 10.1038/ismej.2014.81 24858784 PMC4260704

[B17] Daims H. Lebedeva E. V. Pjevac P. Han P. Herbold C. Albertsen M. (2015). Complete nitrification by Nitrospira bacteria. *Nature* 528 504–509. 10.1038/nature16461 26610024 PMC5152751

[B18] Davidson E. A. de Araújo A. C. Artaxo P. Balch J. K. Brown I. F. Bustamante M. M. C. (2012). The Amazon basin in transition. *Nature* 481 321–328. 10.1038/nature10717 22258611

[B19] Doxey A. C. Kurtz D. A. Lynch M. D. Sauder L. A. Neufeld J. D. (2014). Aquatic metagenomes implicate Thaumarchaeota in global cobalamin production. *ISME J.* 9 461–471. 10.1038/ismej.2014.142 25126756 PMC4303638

[B20] Draper F. C. Roucoux K. H. Lawson I. T. Mitchard E. T. A. Coronado E. N. H. Lähteenoja O. (2014). The distribution and amount of carbon in the largest peatland complex in Amazonia. *Environ. Res. Lett.* 9:124017. 10.1088/1748-9326/9/12/124017

[B21] Drummond J. T. Matthews R. G. (1994). Nitrous oxide degradation by cobalamin-dependent methionine synthase: characterization of the reactants and products in the inactivation reaction. *Biochemistry* 33 3732–3741. 10.1021/bi00178a033 8142373

[B22] Edgar R. C. (2010). Search and clustering orders of magnitude faster than BLAST. *Bioinformatics* 26 2460–2461. 10.1093/bioinformatics/btq461 20709691

[B23] Elberling B. Askaer L. Jørgensen C. J. Joensen H. P. Kühl M. Glud R. N. (2011). Linking soil O_2_. CO_2_, and CH_4_ concentrations in a wetland soil: implications for CO_2_ and CH_4_ fluxes. *Environ. Sci. Technol.* 45 3393–3399. 10.1021/es103540k 21413790

[B24] Ermler U. Grabarse W. Shima S. Goubeaud M. Thauer R. K. (1997). Crystal structure of methyl-coenzyme M reductase: the key enzyme of biological methane formation. *Science* 278 1457–1462. 10.1126/science.278.5342.1457 9367957

[B25] Ettwig K. F. Butler M. K. Le Paslier D. Pelletier E. Mangenot S. Kuypers M. M. M. (2010). Nitrite-driven anaerobic methane oxidation by oxygenic bacteria. *Nature* 464 543–548. 10.1038/nature08883 20336137

[B26] Fetzer S. Conrad R. (1993). Effect of redox potential on methanogenesis by *Methanosarcina barkeri*. *Arch. Microbiol.* 160 108–113. 10.1007/BF00288711

[B27] Finn D. R. Ziv-El M. Haren J. V. Park J. G. Pasquel J. D. A. Muñoz J. D. U. (2020). Methanogens and methanotrophs show nutrient-dependent community assemblage patterns across tropical peatlands of the Pastaza-Marañón basin, Peruvian Amazonia. *Front. Microbiol.* 11:746. 10.3389/fmicb.2020.00746 32390985 PMC7193774

[B28] Gaucher E. A. Miyamoto M. M. Benner S. A. (2001). Function–structure analysis of proteins using covarion-based evolutionary approaches: elongation factors. *Proc. Natl. Acad. Sci. U.S.A.* 98 548–552. 10.1073/pnas.98.2.548 11209054 PMC14624

[B29] Gumbricht T. Cuesta R. M. R. Verchot L. Herold M. Wittmann F. Householder E. (2017). An expert system model for mapping tropical wetlands and peatlands reveals South America as the largest contributor. *Glob. Change Biol.* 23 3581–3599. 10.1111/gcb.13689 28295834

[B30] Hall S. J. Matson P. A. (1999). Nitrogen oxide emissions after nitrogen additions in tropical forests. *Nature* 400 152–155. 10.1038/22094

[B31] Hall S. J. Matson P. A. (2003). Nutrient status of tropical rain forests influences soil N dynamics after N additions. *Ecol. Monogr.* 73 107–129.

[B32] Haroon M. F. Hu S. Shi Y. Imelfort M. Keller J. Hugenholtz P. (2013). Anaerobic oxidation of methane coupled to nitrate reduction in a novel archaeal lineage. *Nature* 500:567. 10.1038/nature12375 23892779

[B33] Herbold C. W. Pelikan C. Kuzyk O. Hausmann B. Angel R. Berry D. (2015). A flexible and economical barcoding approach for highly multiplexed amplicon sequencing of diverse target genes. *Front. Microbiol.* 6:8966. 10.3389/fmicb.2015.00731 26236305 PMC4503924

[B34] Hirano S. Matsumoto N. Morita M. Sasaki K. Ohmura N. (2013). Electrochemical control of redox potential affects methanogenesis of the hydrogenotrophic methanogen Methanothermobacter thermautotrophicus. *Lett. Appl. Microbiol.* 56 315–321. 10.1111/lam.12059 23413966

[B35] Höfferle Š Nicol G. W. Pal L. Hacin J. Prosser J. I Mandić-Mulec I. (2010). Ammonium supply rate influences archaeal and bacterial ammonia oxidizers in a wetland soil vertical profile. *FEMS Microbiol. Ecol.* 74 302–315. 10.1111/j.1574-6941.2010.00961.x 21039647

[B36] Holmes M. E. Chanton J. P. Tfaily M. M. Ogram A. (2015). CO_2_ and CH_4_ isotope compositions and production pathways in a tropical peatland. *Global Biogeochem. Cycles* 29 1–18. 10.1002/2014GB004951

[B37] Houlton B. Z. Sigman D. M. Hedin L. O. (2006). Isotopic evidence for large gaseous nitrogen losses from tropical rainforests. *Proc. Natl. Acad. Sci. U.S.A.* 103 8745–8750. 10.1073/pnas.0510185103 16728510 PMC1469773

[B38] Houlton B. Z. Wang Y.-P. Vitousek P. M. Field C. B. (2008). A unifying framework for dinitrogen fixation in the terrestrial biosphere. *Nature* 454 327–330. 10.1038/nature07028 18563086

[B39] Hu B. L. Shen L. D. Lian X. Zhu Q. Liu S. Huang Q. (2014). Evidence for nitrite-dependent anaerobic methane oxidation as a previously overlooked microbial methane sink in wetlands. *Proc. Natl. Acad. Sci. U.S.A.* 111 4495–4500. 10.1073/pnas.1318393111 24616523 PMC3970540

[B40] Jackson C. R. Liew K. C. Yule C. M. (2008). Structural and functional changes with depth in microbial communities in a tropical Malaysian peat swamp forest. *Microb. Ecol.* 57 402–412. 10.1007/s00248-008-9409-4 18548182

[B41] Jetten M. (1992). Methanogenesis from acetate: a comparison of the acetate metabolism in *Methanothrix soehngenii* and *Methanosarcina* spp. *FEMS Microbiol. Lett.* 88 181–197. 10.1016/0378-1097(92)90802-U

[B42] Jugsujinda A. DeLaune R. D. Lindau C. W. (2008). Influence of nitrate on methane production and oxidation in flooded soil. *Commun. Soil Sci. Plant Anal.* 26 2449–2459. 10.1080/00103629509369459

[B43] Kanokratana P. Uengwetwanit T. Rattanachomsri U. Bunterngsook B. Nimchua T. Tangphatsornruang S. (2010). Insights into the phylogeny and metabolic potential of a primary tropical peat swamp forest microbial community by metagenomic analysis. *Microbiol. Ecol.* 61 518–528. 10.1007/s00248-010-9766-7 21057783

[B44] Kirschke S. Bousquet P. Ciais P. Saunois M. Canadell J. G. Dlugokencky E. J. (2013). Three decades of global methane sources and sinks. *Nat. Geosci.* 6 813–823. 10.1038/ngeo1955

[B45] Klüber H. D. Conrad R. (1998a). Effects of nitrate, nitrite, NO and N_2_O on methanogenesis and other redox processes in anoxic rice field soil. *FEMS Microbiol. Ecol.* 25 301–318.

[B46] Klüber H. D. Conrad R. (1998b). Inhibitory effects of nitrate, nitrite, NO and N_2_O on methanogenesis by *Methanosarcina barkeri* and *Methanobacterium bryantii*. *FEMS Microbiol. Ecol.* 25 331–339. 10.1111/j.1574-6941.1998.tb00484.x

[B47] Lähteenoja O. Ruokolainen K. Schulman L. Alvarez J. (2009). Amazonian floodplains harbour minerotrophic and ombrotrophic peatlands. *Catena* 79 140–145. 10.1016/j.catena.2009.06.006

[B48] Lawson I. T. Jones T. D. Kelly T. J. Coronado E. N. H. Roucoux K. H. (2014). The geochemistry of Amazonian peats. *Wetlands* 34 905–915. 10.1007/s13157-014-0552-z

[B49] Liu B. Frostegård Å Bakken L. R. Bailey M. (2014). Impaired reduction of N_2_O to N_2_ in acid soils is due to a posttranscriptional interference with the expression of nosZ. *mBio* 5 e1383–14. 10.1128/mBio.01383-14 24961695 PMC4073493

[B50] Liu B. Mørkved P. T. Frostegård Å Bakken L. R. (2010). Denitrification gene pools, transcription and kinetics of NO, N_2_O and N_2_ production as affected by soil pH. *FEMS Microbiol. Ecol.* 72 407–417. 20370831 10.1111/j.1574-6941.2010.00856.x

[B51] Manzoni S. Trofymow J. A. Jackson R. B. Porporato A. (2010). Stoichiometric controls on carbon, nitrogen, and phosphorus dynamics in decomposing litter. *Ecol. Monogr.* 80 89–106.

[B52] Matthews R. G. (2001). Cobalamin-dependent methyltransferases. *Acc. Chem. Res.* 34 681–689. 10.1021/ar0000051 11513576

[B53] Min H. Zinder S. H. (1989). Kinetics of acetate utilization by two thermophilic acetotrophic methanogens: *Methanosarcina* sp. strain CALS-1 and *Methanothrix* sp. strain CALS-1. *Appl. Environ. Microbiol.* 55 488–491. 16347856 10.1128/aem.55.2.488-491.1989 PMC184136

[B54] Noll M. Matthies D. Frenzel P. Derakshani M. Liesack W. (2005). Succession of bacterial community structure and diversity in a paddy soil oxygen gradient. *Environ. Microbiol.* 7 382–395. 10.1111/j.1462-2920.2005.00700.x 15683399

[B55] Ondov B. D. Bergman N. H. Phillippy A. M. (2011). Interactive metagenomic visualization in a web browser. *BMC Bioinform.* 12:385. 10.1186/1471-2105-12-385 21961884 PMC3190407

[B56] Orellana L. H. Rodriguez-R L. M. Higgins S. Chee-Sanford J. C. Sanford R. A. Ritalahti K. M. (2014). Detecting nitrous oxide reductase (nosZ) genes in soil metagenomes: method development and implications for the nitrogen cycle. *mBio* 5 e1193–14. 10.1128/mBio.01193-14 24895307 PMC4049103

[B57] Palmer K. Horn M. A. (2015). Denitrification activity of a remarkably diverse fen denitrifier community in Finnish lapland is N-oxide limited. *PLoS One* 10:e0123123. 10.1371/journal.pone.0123123 25860353 PMC4393310

[B58] Parker R. J. Boesch H. McNorton J. Comyn-Platt E. Gloor M. Wilson C. (2018). Evaluating year-to-year anomalies in tropical wetland methane emissions using satellite CH_4_ observations. *Remote Sens. Environ.* 211 261–275. 10.1016/j.rse.2018.02.011

[B59] Penton C. R. St Louis D. Pham A. Cole J. R. Wu L. Luo Y. (2015). Denitrifying and diazotrophic community responses to artificial warming in permafrost and tallgrass prairie soils. *Front. Microbiol.* 6:439. 10.3389/fmicb.2015.00746 26284038 PMC4523034

[B60] Pett-Ridge J. Silver W. L. Firestone M. K. (2006). Redox fluctuations frame microbial community impacts on N-cycling rates in a humid tropical forest soil. *Biogeochemistry* 81 95–110. 10.1007/s10533-006-9032-8

[B61] Philippot L. Andersson S. G. E. Battin T. J. Prosser J. I. Schimel J. P. Whitman W. B. (2010). The ecological coherence of high bacterial taxonomic ranks. *Nat. Methods* 8 523–529. 10.1038/nrmicro2367 20531276

[B62] Price M. N. Dehal P. S. Arkin A. P. (2009). FastTree: computing large minimum evolution trees with profiles instead of a distance matrix. *Mol. Biol. Evol.* 26 1641–1650. 10.1093/molbev/msp077 19377059 PMC2693737

[B63] Puglisi E. Zaccone C. Cappa F. Cocconcelli P. S. Shotyk W. Trevisan M. (2014). Changes in bacterial and archaeal community assemblages along an ombrotrophic peat bog profile. *Biol. Fertil. Soils* 50 815–826. 10.1007/s00374-014-0902-2

[B64] Sakabe A. Itoh M. Hirano T. Kusin K. (2018). Ecosystem-scale methane flux in tropical peat swamp forest in Indonesia. *Glob. Change Biol.* 24 5123–5136. 10.1111/gcb.14410 30175421

[B65] Saraiva L. M. Vicente J. B. Teixeira M. (2004). The role of the flavodiiron proteins in microbial nitric oxide detoxification. *Adv Microb Physiol.* 49 77–129. 15518829 10.1016/S0065-2911(04)49002-X

[B66] Segarra K. E. A. Schubotz F. Samarkin V. Yoshinaga M. Y. Hinrichs K.-U. Joye S. B. (2015). High rates of anaerobic methane oxidation in freshwater wetlands reduce potential atmospheric methane emissions. *Nat. Commun.* 6:7477. 10.1038/ncomms8477 26123199

[B67] Senga Y. Hiroki M. Nakamura Y. Watarai Y. Watanabe Y. Nohara S. (2010). Vertical profiles of DIN, DOC, and microbial activities in the wetland soil of Kushiro Mire, northeastern Japan. *Limnology* 12 17–23. 10.1007/s10201-010-0316-2

[B68] Senga Y. Hiroki M. Terui S. Nohara S. (2015). Variation in microbial function through soil depth profiles in the Kushiro Wetland, northeastern Hokkaido. *Japan. Ecol. Res.* 30 563–572. 10.1007/s11284-015-1257-3

[B69] Steinberg L. M. Regan J. M. (2008). Phylogenetic comparison of the methanogenic communities from an acidic, oligotrophic fen and an anaerobic digester treating municipal wastewater sludge. *Appl. Environ. Microbiol.* 74 6663–6671. 10.1128/AEM.00553-08 18776026 PMC2576706

[B70] Steinberg L. M. Regan J. M. (2009). mcrA-targeted real-time quantitative pcr method to examine methanogen communities. *Appl. Environ. Microbiol.* 75 4435–4442. 10.1128/AEM.02858-08 19447957 PMC2704849

[B71] Stocker B. D. Roth R. Joos F. Spahni R. Steinacher M. Zaehle S. (2013). Multiple greenhouse-gas feedbacks from the land biosphere under future climate change scenarios. *Nat. Clim. Change* 3 666–672. 10.1038/nclimate1864

[B72] Stone M. M. Kan J. Plante A. F. (2015). Parent material and vegetation influence bacterial community structure and nitrogen functional genes along deep tropical soil profiles at the Luquillo Critical Zone Observatory. *Soil Biol. Biochem.* 80 273–282. 10.1016/j.soilbio.2014.10.019

[B73] Stumm W. Morgan J. J. eds (2012). *Aquatic Chemistry*, 3rd Edn. Hoboken NJ: John Wiley & Sons.

[B74] Taketani R. G. Yoshiura C. A. Dias A. C. F. Andreote F. D. Tsai S. M. (2010). Diversity and identification of methanogenic archaea and sulphate-reducing bacteria in sediments from a pristine tropical mangrove. *Antonie Van Leeuwenhoek* 97 401–411. 10.1007/s10482-010-9422-8 20195901

[B75] Takeuchi M. Yoshioka H. Seo Y. Tanabe S. Tamaki H. Kamagata Y. (2011). A distinct freshwater-adapted subgroup of ANME-1 dominates active archaeal communities in terrestrial subsurfaces in Japan. *Environ. Microbiol.* 13 3206–3218. 10.1111/j.1462-2920.2011.02517.x 21651687

[B76] Tamaki H. Wright C. L. Li X. Lin Q. Hwang C. Wang S. (2011). Analysis of 16S rRNA amplicon sequencing options on the Roche/454 Next-Generation Titanium sequencing platform. *PLoS One* 6:e25263. 10.1371/journal.pone.0025263 21966473 PMC3179495

[B77] Tang A. C. I. Stoy P. C. Hirata R. Musin K. K. Aeries E. B. Wenceslaus J. (2018a). Eddy covariance measurements of methane flux at a tropical peat forest in Sarawak. *Malaysian Borneo. Geophys. Res. Lett.* 45 4390–4399. 10.1029/2017gl076457

[B78] Tang Y. Yu G. Zhang X. Wang Q. Ge J. Liu S. (2018b). Changes in nitrogen-cycling microbial communities with depth in temperate and subtropical forest soils. *Appl. Soil Ecol.* 124 218–228. 10.1016/j.apsoil.2017.10.029

[B79] Teh Y. A. Murphy W. A. Berrio J.-C. Boom A. Page S. E. (2017). Seasonal variability in methane and nitrous oxide fluxes from tropical peatlands in the western Amazon basin. *Biogeosciences* 14 3669–3683. 10.5194/bg-14-3669-2017

[B80] Valenzuela E. I. Prieto-Davó A. López-Lozano N. E. Hernández-Eligio A. Vega-Alvarado L. Juárez K. (2017). Anaerobic methane oxidation driven by microbial reduction of natural organic matter in a tropical wetland. *Appl. Environ. Microbiol.* 83 1–40. 10.1128/AEM.00645-17 28341676 PMC5440706

[B81] Van Haren J. L. M. Cadillo-Quiroz H. (2016). *Controls on Tree Species Stem Transport and Emission of Methane from Tropical Peatlands.* Washington, DC: American Geophysical Union.

[B82] van Kessel M. A. H. J. Speth D. R. Albertsen M. Nielsen P. H. Op den Camp H. J. Kartal B. (2015). Complete nitrification by a single microorganism. *Nature* 528 555–559. 10.1038/nature16459 26610025 PMC4878690

[B83] Verchot L. V. Davidson E. A. Cattânio J. H. Ackerman I. L. (2000). Land-use change and biogeochemical controls of methane fluxes in soils of Eastern Amazonia. *Ecosystems* 3 41–56. 10.1007/s100210000009

[B84] Wang Q. Quensen J. F. Fish J. A. Lee T. K. Sun Y. Tiedje J. M. (2013). Ecological patterns of nifH genes in four terrestrial climatic zones explored with targeted metagenomics using FrameBot, a New Informatics Tool. *mBio* 4 e00592–13. 10.1128/mBio.00592-13 24045641 PMC3781835

[B85] Wang X. Helgason B. Westbrook C. Bedard-Haughn A. (2016). Effect of mineral sediments on carbon mineralization, organic matter composition and microbial community dynamics in a mountain peatland. *Soil Biol. Biochem.* 103 16–27. 10.1016/j.soilbio.2016.07.025

[B86] Watanabe T. Wang G. Taki K. Ohashi Y. Kimura M. Asakawa S. (2010). Vertical changes in bacterial and archaeal communities with soil depth in Japanese paddy fields. *Soil Sci. Plant Nutr.* 56 705–715. 10.1111/j.1747-0765.2010.00511.x

[B87] Weber E. B. Lehtovirta-Morley L. E. Prosser J. I. Gubry-Rangin C. (2015). Ammonia oxidation is not required for growth of Group 1.1c soil Thaumarchaeota. *FEMS Microbiol. Ecol.* 91:fiaa256. 10.1093/femsec PMC4399444 25764563

[B88] Whalen S. C. (2005). Biogeochemistry of methane exchange between natural wetlands and the atmosphere. *Environ. Eng. Sci.* 22 73–94.

[B89] Whiticar M. J. (1999). Carbon and hydrogen isotope systematics of bacterial formation and oxidation of methane. *Chem. Geol.* 161 291–314.

[B90] Winton R. S. Flanagan N. Richardson C. J. (2017). Neotropical peatland methane emissions along a vegetation and biogeochemical gradient. *PLoS One* 12:e0187019. 10.1371/journal.pone.0187019 29053738 PMC5650183

[B91] Wong G. X. Hirata R. Hirano T. Kiew F. Aeries E. B. Musin K. K. (2018). Micrometeorological measurement of methane flux above a tropical peat swamp forest. *Agric. For. Meteorol.* 25 353–361. 10.1016/j.agrformet.2018.03.025

[B92] Wong G. X. Hirata R. Hirano T. Kiew F. Aeries E. B. Musin K. K. (2020). How do land use practices affect methane emissions from tropical peat ecosystems? *Agric. For. Meteorol.* 28:107869. 10.1016/j.agrformet.2019.107869

[B93] Yang S. Liebner S. Alawi M. Ebenhöh O. Wagner D. (2014). Taxonomic database and cut-off value for processing mcrA gene 454 pyrosequencing data by MOTHUR. *J. Microbiol. Methods* 103 3–5. 10.1016/j.mimet.2014.05.006 24858450

[B94] Yao H. Gao Y. Nicol G. W. Campbell C. D. Prosser J. I. Zhang L. (2011). Links between ammonia oxidizer community structure, abundance, and nitrification potential in acidic soils. *Appl. Environ. Microbiol.* 77 4618–4625. 10.1128/AEM.00136-11 21571885 PMC3127715

[B95] Yarnes C. (2013). δ13C and δ2H measurement of methane from ecological and geological sources by gas chromatography/combustion/pyrolysis isotope-ratio mass spectrometry. *Rapid Commun. Mass Spectrom.* 27 1036–1044. 10.1002/rcm.6549 23592207

[B96] Yu Y. Lee C. Kim J. Hwang S. (2005). Group-specific primer and probe sets to detect methanogenic communities using quantitative real-time polymerase chain reaction. *Biotechnol. Bioeng.* 89 670–679. 10.1002/bit.20347 15696537

[B97] Zhang L.-M. Hu H.-W. Shen J.-P. He J.-Z. (2012). Ammonia-oxidizing archaea have more important role than ammonia-oxidizing bacteria in ammonia oxidation of strongly acidic soils. *ISME J.* 6, 1032–1045. 10.1038/ismej.2011.168 22134644 PMC3329103

[B98] Zhu B. van Dijk G. Fritz C. Smolders A. J. P. Pol A. Jetten M. S. M. (2012). Anaerobic oxidization of methane in a minerotrophic peatland: enrichment of nitrite-dependent methane-oxidizing bacteria. *Appl. Environ. Microbiol.* 78 8657–8665. 10.1128/AEM.02102-12 23042166 PMC3502929

