# Supplementary material for: Microbial Communities and Interactions of Nitrogen Oxides With Methanogenesis in Diverse Peatlands of the Amazon Basin
Source: Front Microbiol. 2021 Jun 29;12:659079. doi: 10.3389/fmicb.2021.659079 (PMC8276178; doi:10.3389/fmicb.2021.659079)
Supplement: Supplementary file 1 [file Data_Sheet_1.pdf]

Supplementary Material

Microbial Communities and Interactions of Nitrogen Oxides With Methanogenesis in Diverse Peatlands of the Amazon Basin

S. Buessecker, Z. Zamora, A. F. Sarno, D. R. Finn, A. M. Hoyt, J. van Haren, J. D. Urquiza Muñoz, and H. Cadillo-Quiroz

Correspondence to: H. Cadillo-Quiroz (hinsby@asu.edu)

1 Supplementary Discussion

Dissolved organic carbon (DOC) profiles. DOC has emerged as an important predictor of CH_4 production in wetland soils (Liu et al., 2012; Morrissey et al., 2013). In comparison, DOC concentrations obtained in this study are at the lower end of values previously reported from Borneo peats (Gandois et al., 2014). All concentration profiles appear stable along soil depth with no indication of sinks. These would conceivably occur in more shallow regions where the fraction of refractory soil carbon is less prevalent (Artz et al., 2006). A DOC sink due to mineralization is not present based on our data. The DOC pool is comprised of diverse organic molecules characterized by a wide molecular size range, organic acids, humic substances of different aromaticities, and protein groups. These compounds may vary with depth even though the bulk concentration may be stagnant. Extracellular enzymes and humic substances may play a crucial role in diverting electron flow from methanogenesis, given the thermodynamic preference over CO_2 (Heitmann et al., 2007; Knorr and Blodau, 2009), that would ultimately suppress CH_4 production. Based on our results, we can exclude fluctuations of the total DOC pool to have major effects on carbon mineralization in distinct soil layers, but organic compounds could structurally differ along depth and impact specific microbial activities.

***Xanthobacteriaceae* and Bathyarchaeota are potentially important decomposers of organic matter in tropical peat soils.** The high abundance of *Xanthobacteriaceae* and Bathyarchaeota (Fig. S3) across sites is likely related to a putative role as degraders of peat organic matter. Particularly under O_2-limiting conditions, *Xanthobacteriaceae* have a broad suite of organic substrates, including alkanes, alkenes, (poly)aromatic compounds, thiopenes, organic acids, or xylose and xylan (Zaichikova et al., 2010). Those substrates are common and accumulate in peat soils

(Moers et al., 1990). Bathyarchaeota is an abundant group likely to have been overlooked by the lack of recognition of the phyla in older databases (as in SILVA 115 or priors; (Bai et al., 2018), or because several primers are incapable of amplification (like the *mcrA* set in this study that has 10-18 base pair mismatches; (Evans et al., 2015)). The genetic potential of Bathyarchaeota includes enzymes to hydrolyze plant-derived carbohydrates and detrital proteins, and to produce acetate for energy conservation (Lazar et al., 2016). A putative relationship Bathyarchaeota-NO_x is supported by a report indicating that NO_2^-/NO_3^- transporter proteins, an enzymatic set to support DNRA, were detected in reconstructed Bathyarchaeota metagenomes (Lazar et al., 2016). PC analysis indicates such interactions, where Bathyarchaeota diversity, pH and NO_x concomitantly explain over 50% of data variation along PC 1 (Fig. 2B).

2 Supplementary Figures and Tables

2.1 Supplementary Figures

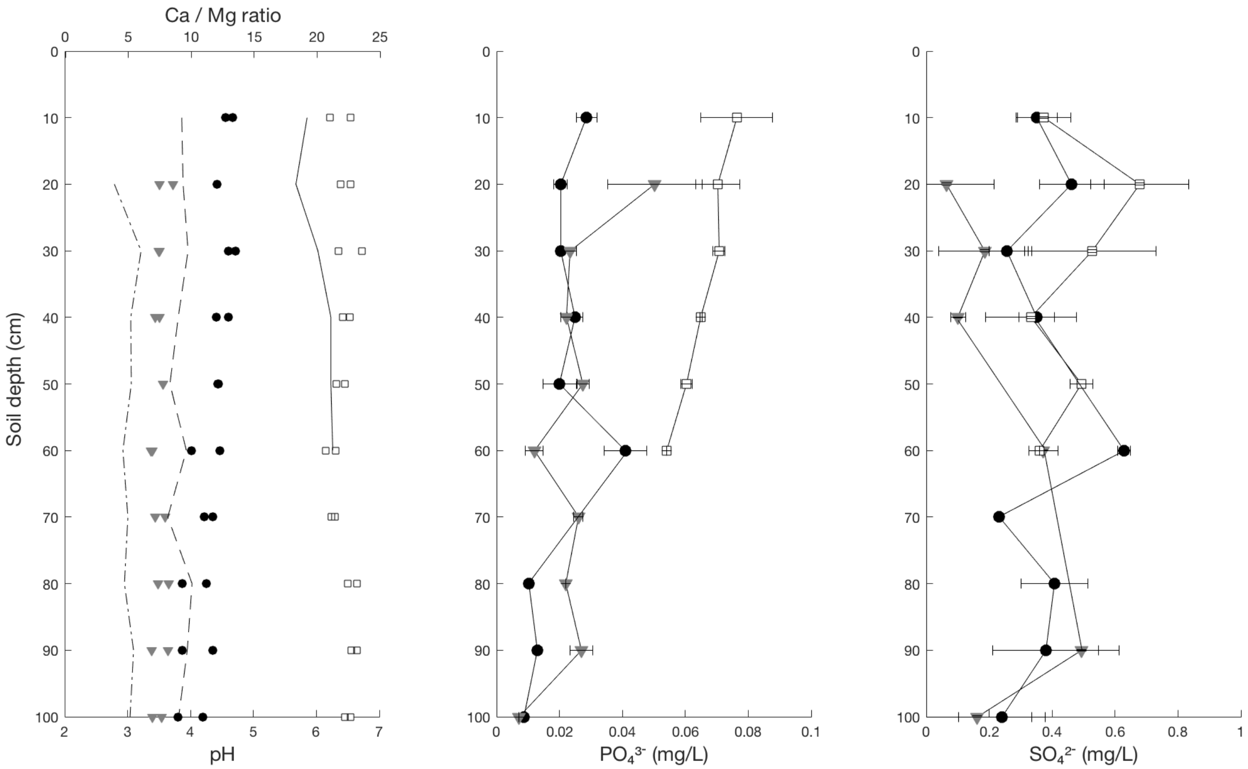

Supplementary Figure 1. Left panel: Ca/Mg ratio in lines for QUI (dashed), BVA (solid), and SJO (point-dashed). pH in symbols for QUI (circles), SJO (triangles), and BVA (squares). Each point is derived from one replicate soil core. **Middle panel:** PO_4^{3-} concentration with data points representing the mean of values from replicate soil cores. **Right panel:** SO_4^{2-} concentration with data points representing the mean of values from replicate soil cores. Error bars denote one *SD* and symbols are consistent with left panel.

Pore water of SJO strongly resembles rainwater composition (Ca/Mg mean of ~8.8, Honório et al., 2010). BVA is likely influenced by groundwater (vertical or lateral water movements) that provides the peat body with minerals. Minerotrophic peatlands have a mineral-derived alkalinity that counteracts acidification caused by the degradation of plant litter and the release of organic acids. Ombrotrophic peatlands are characterized by a low basic cation content that lacks buffer capacity. BVA is a minerotrophic site, where as QUI is transitional minerotrophic-ombrotrophic. SJO is strictly ombrotrophic and the convex shape of this peatland further induces a barrier for groundwater to reach top soil layers (Lähteenoja et al., 2009).

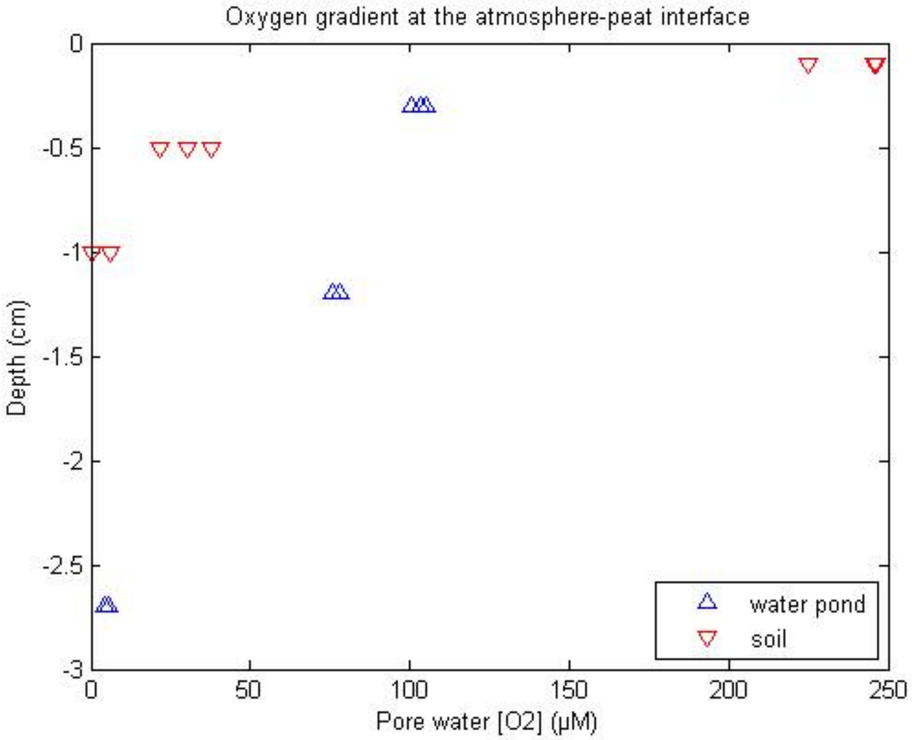

Supplementary Figure 2. Dissolved oxygen concentration in water of saturated soil. Measurements were done using a microelectrode, which was either inserted directly into soft peat (red symbols) or into a shallow (~3 cm) water pond (blue symbols). Measurements were conducted in QUI peatland showing O_2 concentrations drop below 1 μM detection levels after 1 or 2.7 cm depths.

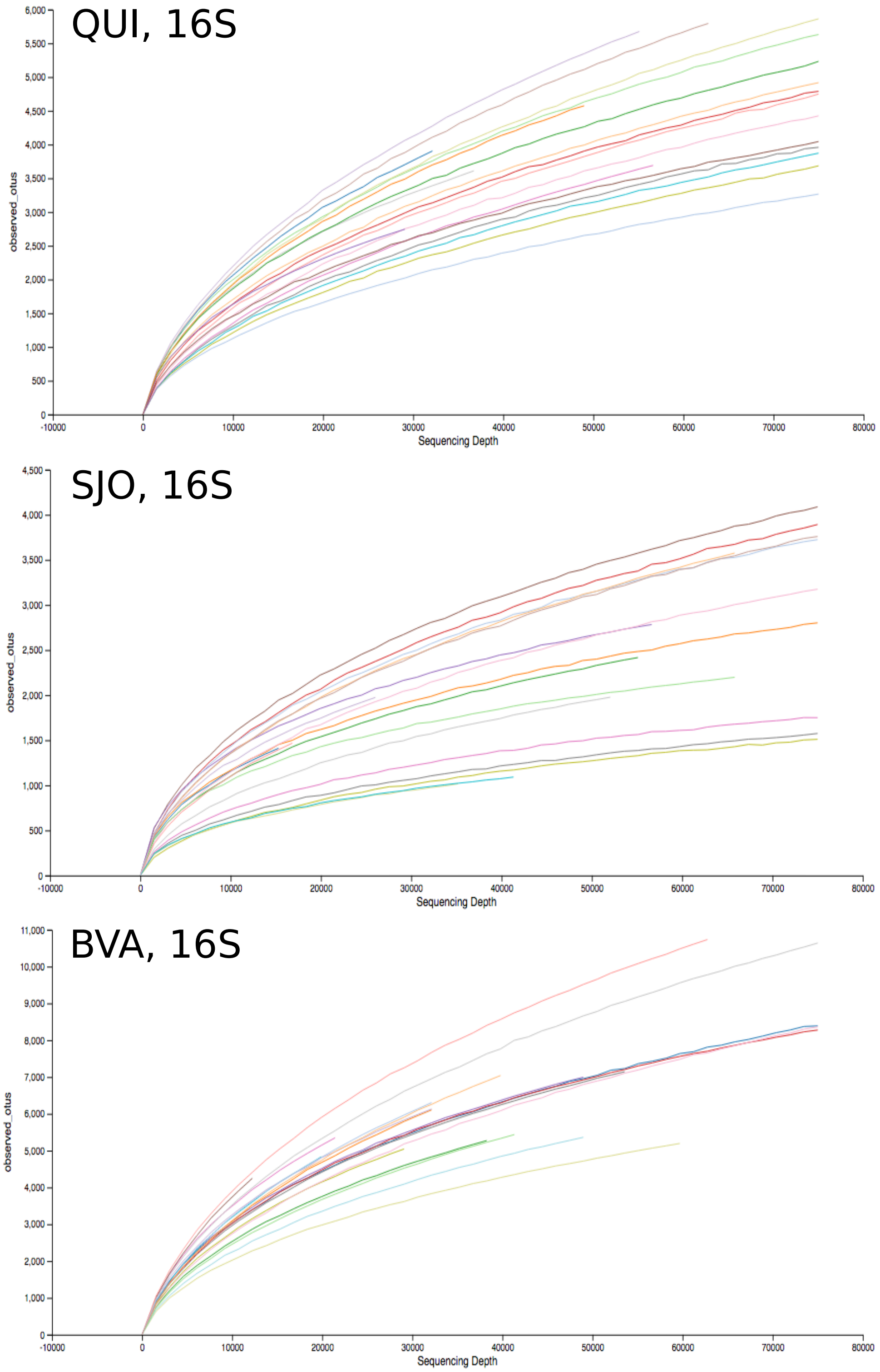

Supplementary Figure 3. Alpha-rarefaction curves for 16S rRNA sequences, used to adjust for variability in library size across samples. Rarefaction curves were used to determine the minimum number of reads per sample used for alpha diversity metrics.

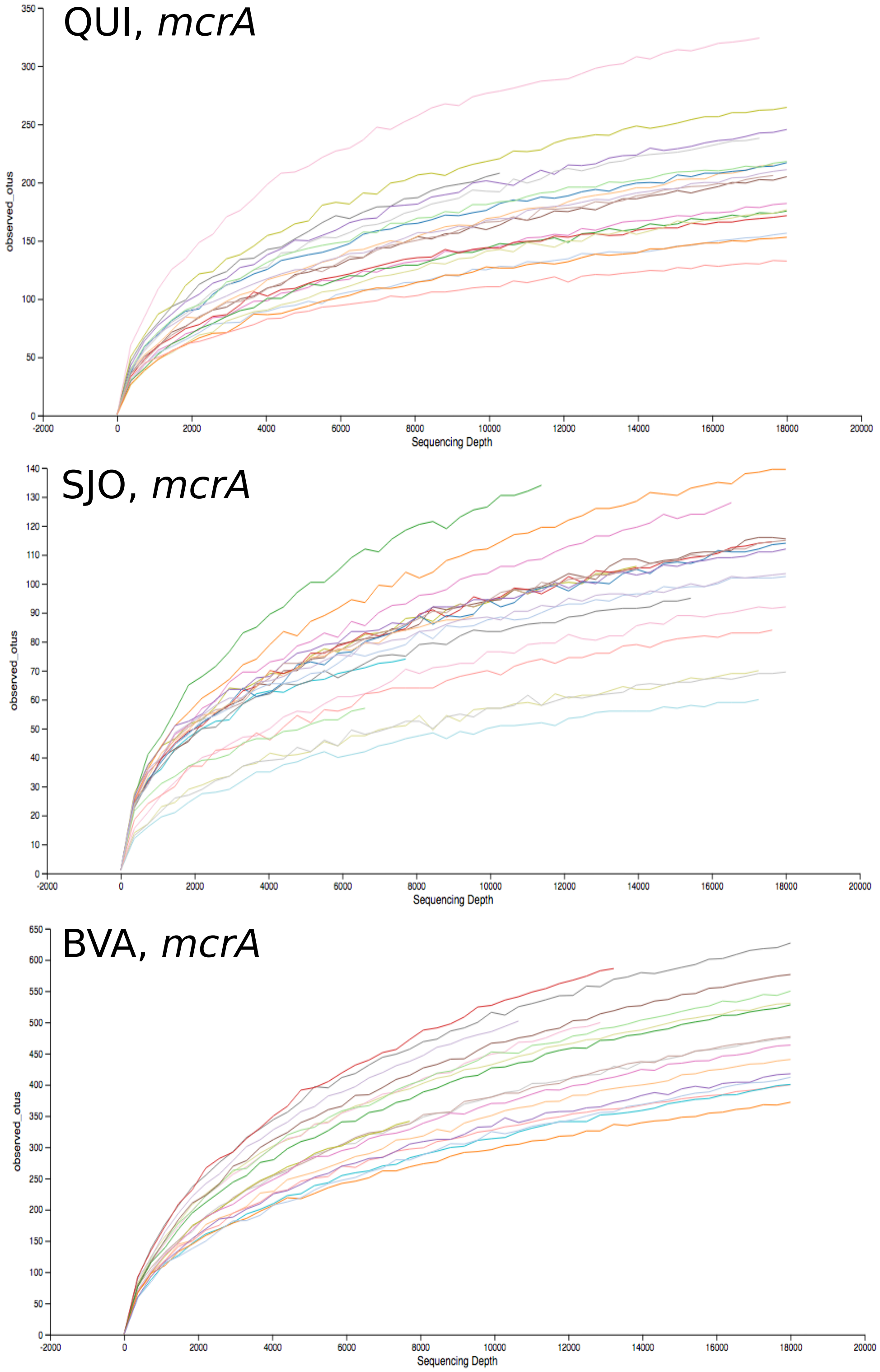

Supplementary Figure 3 continued. Alpha-rarefaction curves for *mcrA* sequences, used to adjust for variability in library size across samples. Rarefaction curves were used to determine the minimum number of reads per sample used for alpha diversity metrics. Samples were rarefied to 20,000 reads per sample (Yang et al., 2017).

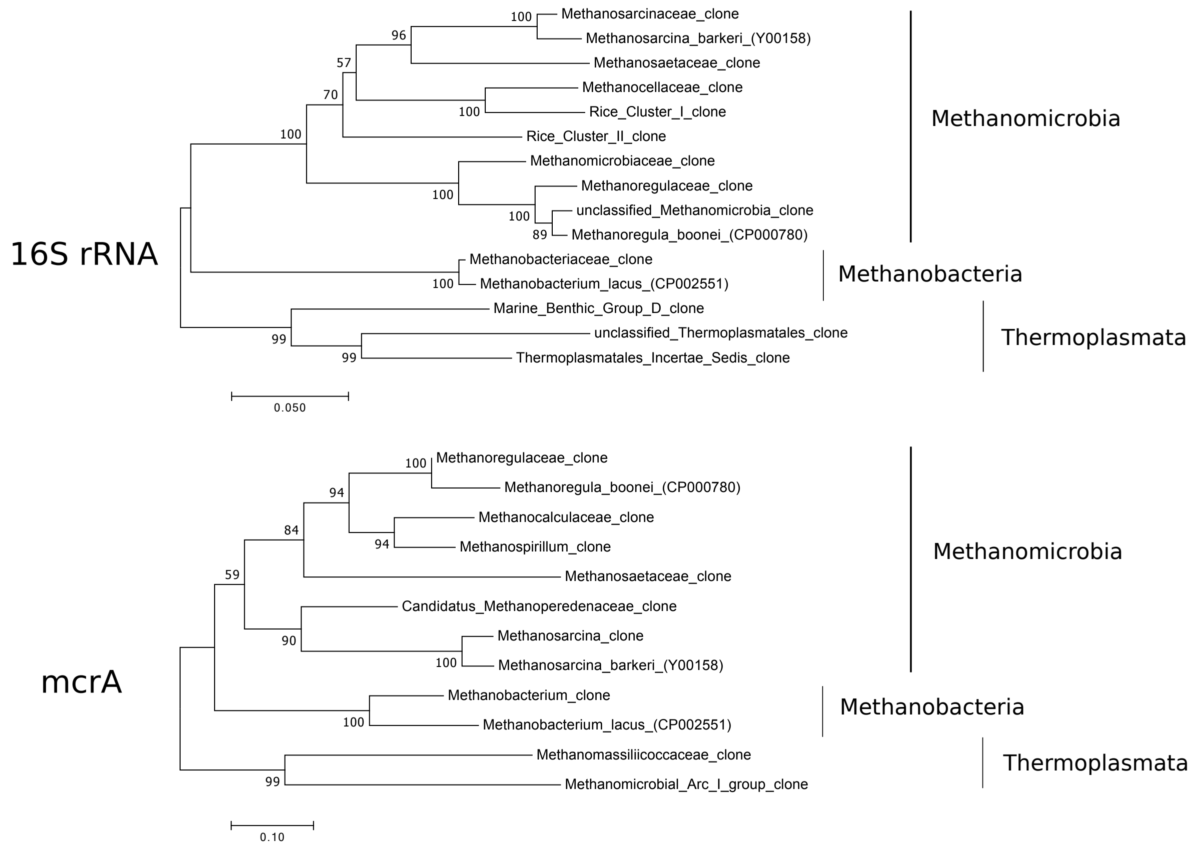

Supplementary Figure 4. 16S rRNA and *mcrA* phylogenetic trees based on the neighbor-joining method and 100 iterations. The trees reflect congruency of classified sequences from 16S and *mcrA* amplicon sequencing.

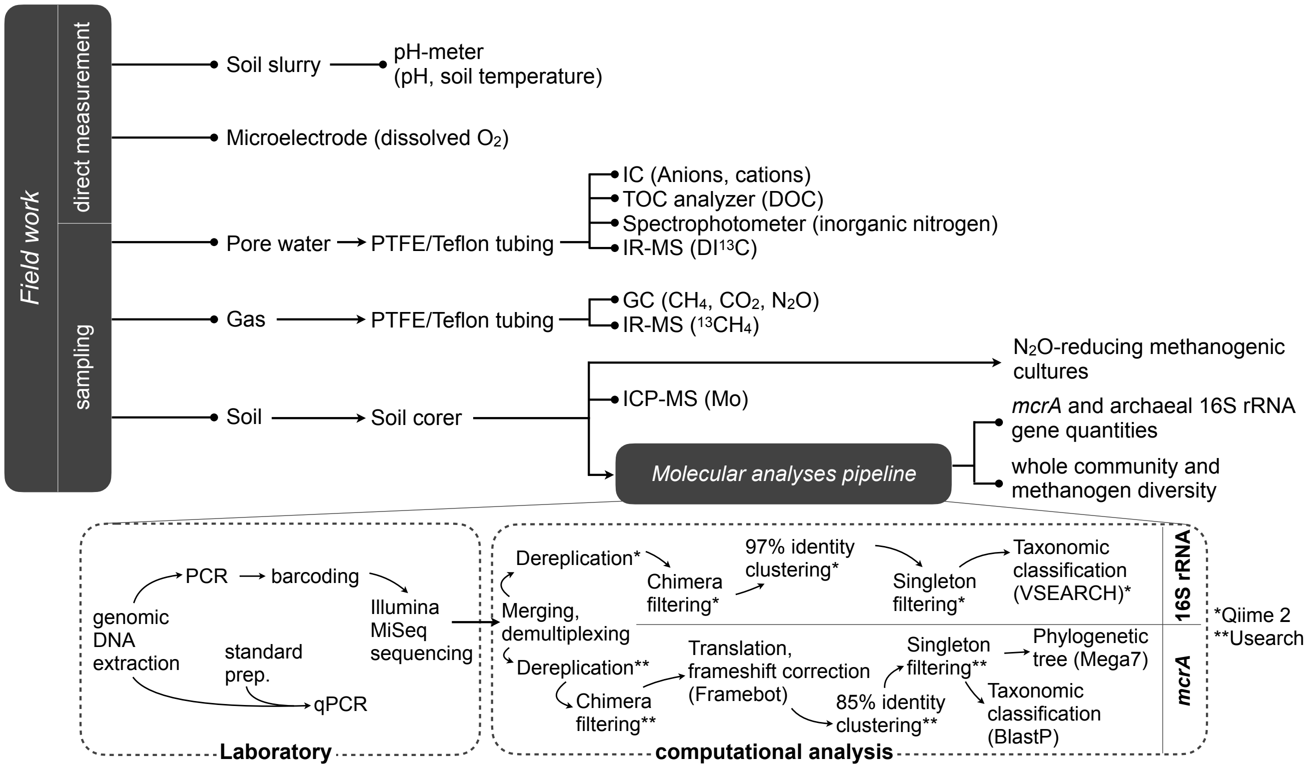

Supplementary Figure 5. Workflow overview including the molecular analysis pipeline.

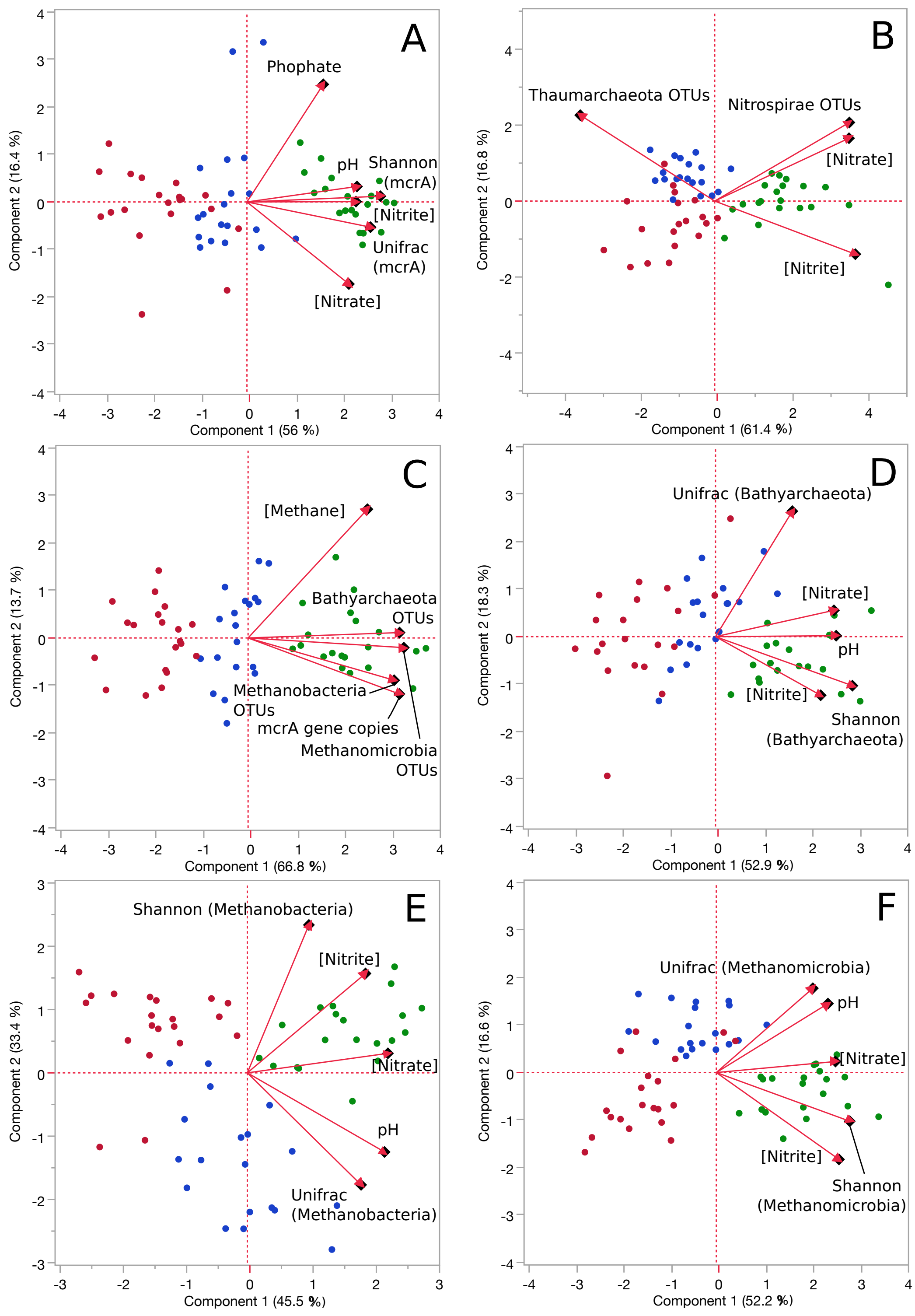

Supplementary Figure 6. Principal component Analysis (PCA) ordination plots of microbial and environmental data from three soil profiles of contrasting Amazon peatlands. Samples were derived from QUI (triangles), SJO (circles) and BVA (squares) with abbreviations as in Fig. 1. Extended environmental variables or microbial groups were evaluated against overall microbial communities variation**.** Figure 3 in main text is identical to panels C and D.

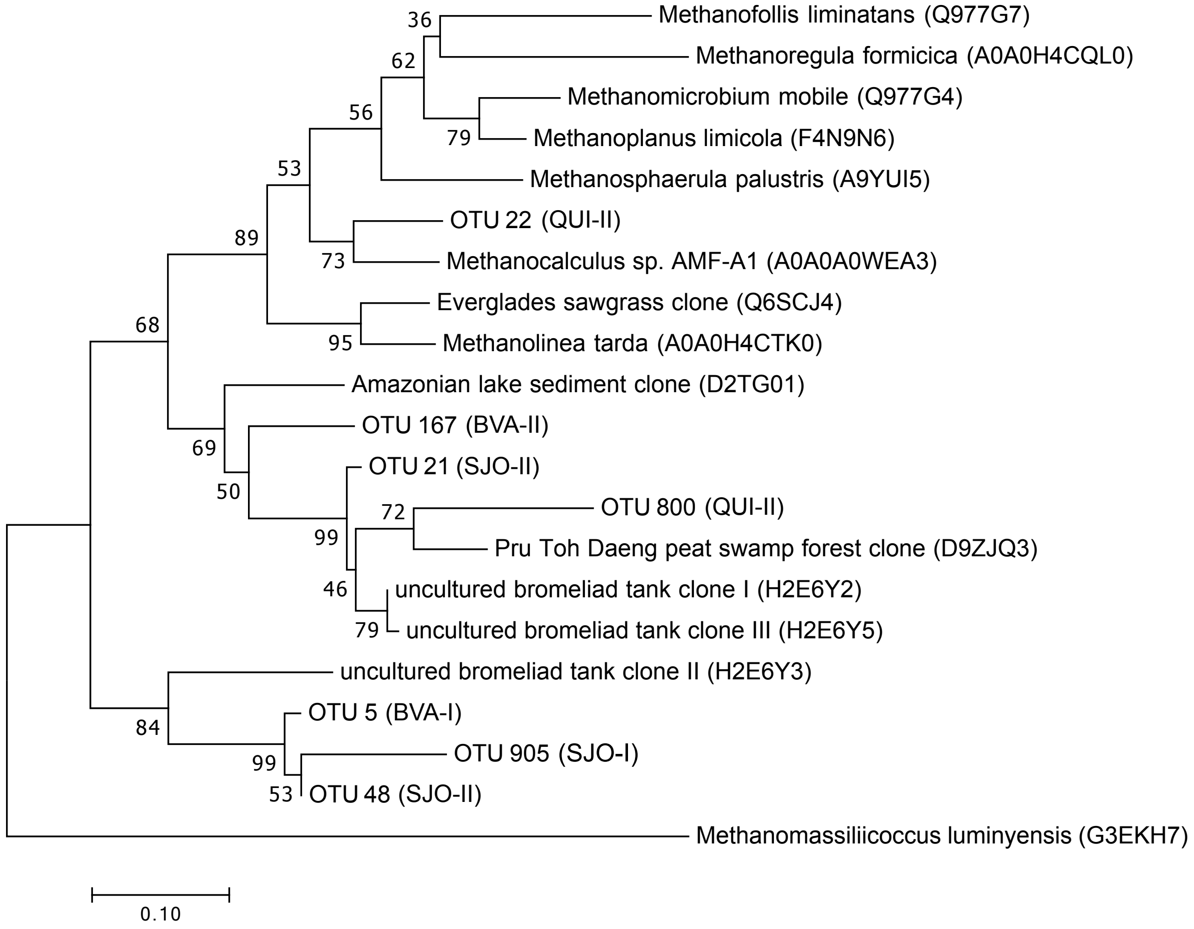

Supplementary Figure 7. Neighbor-joining (1000 iterations) phylogenetic analysis of selected Methanomicrobiales *mcrA* sequences from three Amazon peatlands. Reference sequences were picked from environmental data derived from tropical peatlands, Amazon lakes (Conrad et al., 2010), and tropical vegetation (Goffredi et al., 2011), and from isolates for which the accession number is provided.

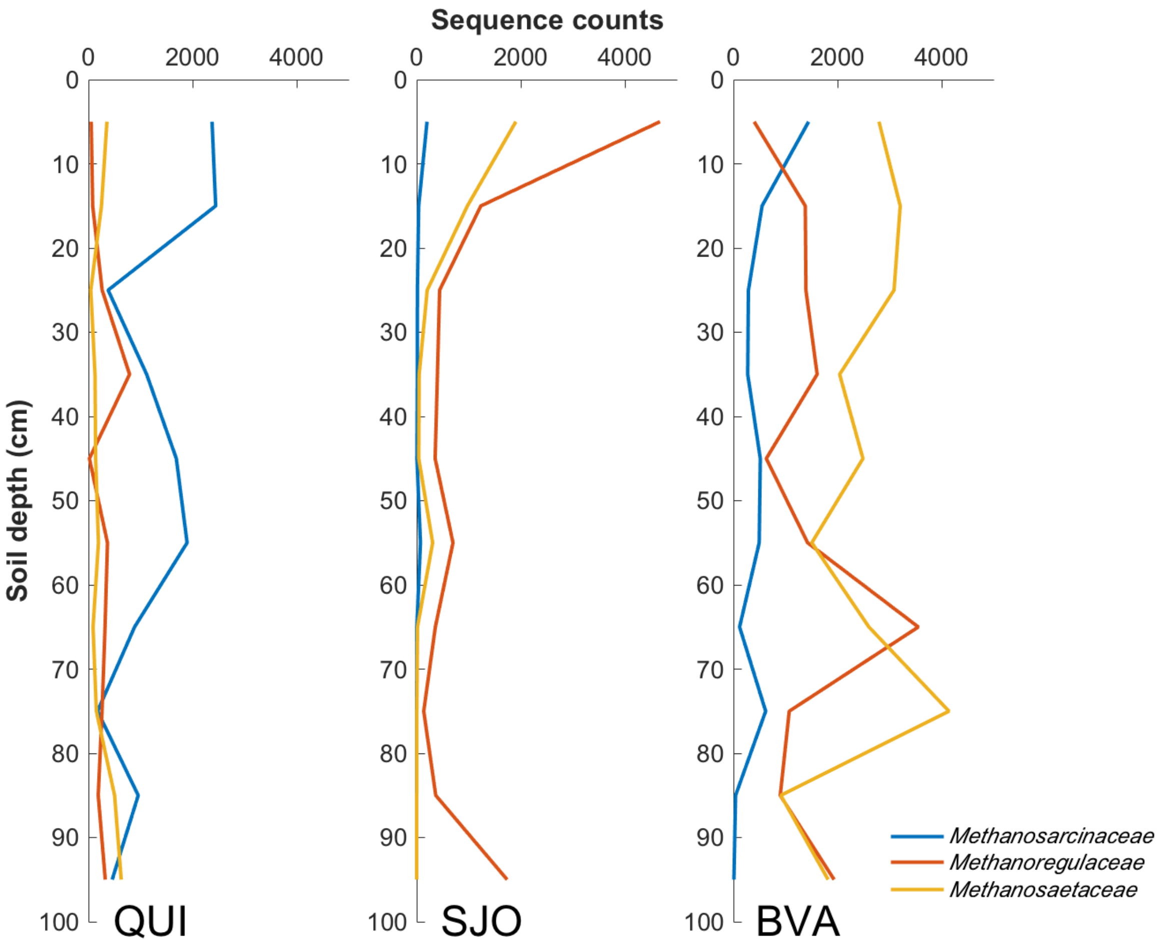

Supplementary Figure 8. Distribution of raw *mcrA* sequence counts along soil profiles. QUI: Quistococha, BVA: Buena Vista and SJO: San Jorge

2.2 Supplementary Tables

Supplementary Table 1. OTU abundances and microbial family metabolic capacity and traits.

Taxon	Core	Mean relative abundance along depth (%)	N metabolic reaction	Potential syntrophy with methanogen	Reference
Acido-bacteriaceae	QUI-1	5.0	N respiration not a dominant feature of this family.		Rosenberg et al., 2014
	QUI-2	3.5			
	SJO-1	8.3			
	SJO-2	6.0			
	BVA-1	0.7			
	BVA-2	0.7			
Solibacteraceae	QUI-1	1.3	N respiration not a dominant feature of this family.		Eichorst et al., 2011
	QUI-2	0.8			
	SJO-1	2.3			
	SJO-2	1.3			
	BVA-1	0.3			
	BVA-2	0.2			
Acido-thermaceae	QUI-1	1.3	Denitrification (*Acidothermus*) not verified in culture.		Barta et al., 2017 Mohagheghi et al., 1986
	QUI-2	0.5			
	SJO-1	1.6			
	SJO-2	1.4			
	BVA-1	0.1			
	BVA-2	0.1			
Bacillaceae	QUI-1	8.4	Denitrification, DNRA and sole N_2O reduction (*Bacillus* and *Geobacillus*). Nitrate reduction verified in culture.		Mandić-Mulec et al., 2015
	QUI-2	2.1			
	SJO-1	0.0			
	SJO-2	0.0			
	BVA-1	0.2			
	BVA-2	0.2			
Paeni-bacillaceae	QUI-1	2.7	Nitrate reduction and N fixation (*Paenibacillus*). Nitrate reduction verified in culture.		Yoon, 2003 Beneduzi et al., 2010
	QUI-2	0.1			
	SJO-1	0.0			
	SJO-2	0.0			
	BVA-1	0.9			
	BVA-2	0.6			

Supplementary Table 1 continued

Taxon	Core	Mean relative abundance along depth (%)	N metabolic reaction	Potential syntrophy with methanogen	Reference
Nitrospiraceae	QUI-1	0.5	Nitrite oxidation (*Nitrospira*), Fe^{2+} oxidation (*Lepto-spririllum*). Nitrite oxidation verified in culture.		Rosenberg et al., 2014
	QUI-2	0.5			
	SJO-1	0.7			
	SJO-2	0.9			
	BVA-1	3.7			
	BVA-2	3.3			
Plancto-mycetaceae	QUI-1	3.0	Anammox (Cand. Anammoxi-microbium), Nitrate reduction (*Planctomyces, Rhodopirellula*). Nitrate reduction verified in culture.		Khramenkov et al., 2013<br>Bondoso et al., 2014
	QUI-2	1.6			
	SJO-1	1.9			
	SJO-2	1.9			
	BVA-1	2.2			
	BVA-2	2.1			
Bradyrhi-zobiaceae	QUI-1	0.1	Nitrite oxidation (*Nitrobacter*), N fixation (*Brady-rhizobium*). N mineralization and nitrate production activity verified in culture.		Zahran, 1999<br>Rosenberg et al., 2014
	QUI-2	0.1			
	SJO-1	0.2			
	SJO-2	0.1			
	BVA-1	0.2			
	BVA-2	0.1			
Hypho-microbiaceae	QUI-1	0.5	Denitrification (*Hypho-microbium*). Nitrate reduction verified in culture.		Martineau et al., 2015
	QUI-2	0.3			
	SJO-1	0.1			
	SJO-2	0.6			
	BVA-1	0.9			
	BVA-2	0.7			
Xantho-bacteraceae	QUI-1	2.3	N fixation.		Rosenberg et al., 2014
	QUI-2	1.7			
	SJO-1	4.1			
	SJO-2	5.4			
	BVA-1	2.0			
	BVA-2	1.9			

Supplementary Table 1 continued

Taxon	Core	Mean relative abundance along depth (%)	N metabolic reaction	Potential syntrophy with methanogen	Reference
Rhodo-spirillaceae	QUI-1	0.5	N fixation (*Azospirillum*), Nitrate reduction (*Azospirillum, Magneto-spirillum*), N_2O reduction (*Azospririllum*). Nitrate reduction verified in culture.		Rosenberg et al., 2014
	QUI-2	0.4			
	SJO-1	0.6			
	SJO-2	0.5			
	BVA-1	0.2			
	BVA-2	0.2			
Alcaligenaceae	QUI-1	0.2	Nitrate reduction (*Candidimonas*), Nitrite reduction (*Alcaligenes*). Nitrate reduction verified in culture.		Rosenberg et al., 2014 Vaz-Moreira et al., 2011
	QUI-2	0.2			
	SJO-1	0.5			
	SJO-2	0.4			
	BVA-1	0.0			
	BVA-2	0.0			
Burk-holderiaceae	QUI-1	0.3	Denitrification. Nitrate reduction verified in culture.		Rosenberg et al., 2014
	QUI-2	1.6			
	SJO-1	18.6			
	SJO-2	11.1			
	BVA-1	0.2			
	BVA-2	0.2			
Coma-monadaceae	QUI-1	0.0	Nitrate reduction (*Comamonas, Brachymonas, Diaphorobacter*). Nitrate reduction verified in culture.		Rosenberg et al., 2014
	QUI-2	0.0			
	SJO-1	0.4			
	SJO-2	0.0			
	BVA-1	1.1			
	BVA-2	1.2			
Neisseriaceae	QUI-1	0.0	Nitrite and NO reduction (*Neisseria*). Nitrite reduction verified in culture.		Rock et al., 2005
	QUI-2	0.0			
	SJO-1	1.9			
	SJO-2	0.0			
	BVA-1	0.2			
	BVA-2	0.3			

Supplementary Table 1 continued

**Taxon**	**Core**	**Mean relative abundance along depth (%)**	**N metabolic reaction**	**Potential syntrophy with methanogen**	**Reference**
Nitroso-monadaceae	QUI-1	0.1	Ammonia oxidation. Verified in culture.		Rosenberg et al., 2014
	QUI-2	0.0			
	SJO-1	0.0			
	SJO-2	0.0			
	BVA-1	0.8			
	BVA-2	0.7			
Desulfu-rellaceae	QUI-1	0.5		H_2 con-sumption and syntrophic association with methanogen? Competition possible.	Miroshnichenko et al., 1998
	QUI-2	0.4			
	SJO-1	0.9			
	SJO-2	0.5			
	BVA-1	0.8			
	BVA-2	0.7			
Syntrophaceae	QUI-1	1.0		Propionate oxidation and syntrophy with methano-gens. Syntrophy studied in cultures.	Lueders et al., 2003
	QUI-2	1.2			
	SJO-1	0.6			
	SJO-2	0.8			
	BVA-1	2.1			
	BVA-2	1.7			
Syntropho-bacteraceae	QUI-1	0.8		Fermenter or syntrophic association with H_2/formate-utilizing partners. Syntrophy studied in cultures.	Rosenberg et al., 2014
	QUI-2	0.6			
	SJO-1	1.1			
	SJO-2	0.8			
	BVA-1	0.4			
	BVA-2	0.3			
Pseudo-monadaceae	QUI-1	2.2	Denitrification (*Pseudomonas*). Nitrate and nitrite reduction verified in culture.		Rosenberg et al., 2014
	QUI-2	1.9			
	SJO-1	0.0			
	SJO-2	0.0			
	BVA-1	1.0			
	BVA-2	1.3			

Supplementary Table 1 continued

**Taxon**	**Core**	**Mean relative abundance along depth (%)**	**N metabolic reaction**	**Potential syntrophy with methanogen**	**Reference**
Spirochaetaceae	QUI-1	0.8		Fermenter with known syntrophic association with methano-gens (*Sphaero-chaeta*). Syntrophy studied in cultures.	Troshina et al., 2015
	QUI-2	0.7			
	SJO-1	0.5			
	SJO-2	0.6			
	BVA-1	1.0			
	BVA-2	1.0			
Opitutaceae	QUI-1	0.1	N fixation (*Diplosphaera*) and nitrate reduction (*Opitutus*). Nitrate reduction verified in culture.	Propionate production (*Opitutus*). Syntrophy not assessed in culture.	Chin et al., 2001
	QUI-2	0.1			
	SJO-1	0.1			
	SJO-2	0.1			
	BVA-1	0.0			
	BVA-2	0.1			

Supplementary Table 2. Spearman's rank multivariate analysis of geochemical parameters (pH and concentration of different species) and selected methanogen taxa. Increasing positive correlation is indicated by blue text color and increasing negative correlation is indicated by red text color. Correlation of a parameter with itself = 1.0.

Quistococha (QUI)	pH	Phosphate	Nitrite	Nitrate	Ammonium	Sulfate	Methanobacteriaceae	Methanoregulaceae	Methanosaetaceae	Methanosarcinaceae	Methanocellaceae	Methanomassiliicoccaceae
pH	1.0000	-0.0301	-0.0840	0.4560	-0.4968	-0.3888	0.1907	-0.3200	-0.4336	0.4939	-0.3586	0.3818
Phosphate	-0.0301	1.0000	-0.0118	-0.2441	0.3215	-0.0392	-0.0514	0.1474	0.3532	-0.0640	-0.0196	-0.2264
Nitrite	-0.0840	-0.0118	1.0000	0.0845	0.1631	-0.1345	0.4523	-0.1629	0.0762	0.3139	-0.0765	0.3098
Nitrate	0.4560	-0.2441	0.0845	1.0000	-0.3187	-0.3320	0.0627	-0.1910	-0.1808	0.0347	-0.2097	0.0853
Ammonium	-0.4968	0.3215	0.1631	-0.3187	1.0000	0.5091	-0.3472	0.3686	0.4532	-0.3578	-0.0966	-0.4121
Sulfate	-0.3888	-0.0392	-0.1345	-0.3320	0.5091	1.0000	-0.0674	0.1930	0.4966	-0.1086	-0.0606	-0.0065
Methanobacteriaceae	0.1907	-0.0514	0.4523	0.0627	-0.3472	-0.0674	1.0000	-0.3189	0.2621	0.7457	-0.0584	0.8491
Methanoregulaceae	-0.3200	0.1474	-0.1629	-0.1910	0.3686	0.1930	-0.3189	1.0000	0.1815	-0.2867	0.1087	-0.4746
Methanosaetaceae	-0.4336	0.3532	0.0762	-0.1808	0.4532	0.4966	0.2621	0.1815	1.0000	-0.1805	0.0793	-0.0388
Methanosarcinaceae	0.4939	-0.0640	0.3139	0.0347	-0.3578	-0.1086	0.7457	-0.2867	-0.1805	1.0000	-0.3540	0.7585
Methanocellaceae	-0.3586	-0.0196	-0.0765	-0.2097	-0.0966	-0.0606	-0.0584	0.1087	0.0793	-0.3540	1.0000	-0.1510
Methanomassiliicoccaceae	0.3818	-0.2264	0.3098	0.0853	-0.4121	-0.0065	0.8491	-0.4746	-0.0388	0.7585	-0.1510	1.0000
San Jorge (SJO)												
pH	1.0000	0.3968	0.0302	-0.0287	-0.1371	0.2855	-0.1593	-0.3097	-0.0479	-0.2067	-0.1164	-0.0454
Phosphate	0.3968	1.0000	0.0339	-0.1553	-0.1813	0.4215	-0.3666	-0.3588	-0.1796	-0.1216	-0.0081	-0.1393
Nitrite	0.0302	0.0339	1.0000	0.0457	-0.4562	-0.6845	0.0284	0.0529	0.2279	0.0262	0.0845	0.0646
Nitrate	-0.0287	-0.1553	0.0457	1.0000	-0.4835	-0.2225	0.0737	0.2166	0.6195	0.7156	0.4142	0.6312
Ammonium	-0.1371	-0.1813	-0.4562	-0.4835	1.0000	0.0822	-0.0870	-0.2831	-0.4339	-0.4213	-0.3646	-0.3895
Sulfate	0.2855	0.4215	-0.6845	-0.2225	0.0822	1.0000	-0.2405	-0.0831	-0.2052	-0.1255	0.1085	0.0106
Methanobacteriaceae	-0.1593	-0.3666	0.0284	0.0737	-0.0870	-0.2405	1.0000	0.3381	0.1431	0.0788	0.0443	0.0312
Methanoregulaceae	-0.3097	-0.3588	0.0529	0.2166	-0.2831	-0.0831	0.3381	1.0000	0.8278	0.7137	0.8891	0.6934
Methanosaetaceae	-0.0479	-0.1796	0.2279	0.6195	-0.4339	-0.2052	0.1431	0.8278	1.0000	0.8978	0.9441	0.9523
Methanosarcinaceae	-0.2067	-0.1216	0.0262	0.7156	-0.4213	-0.1255	0.0788	0.7137	0.8978	1.0000	0.8211	0.9109
Methanocellaceae	-0.1164	-0.0081	0.0845	0.4142	-0.3646	0.1085	0.0443	0.8891	0.9441	0.8211	1.0000	0.8918
Methanomassiliicoccaceae	-0.0454	-0.1393	0.0646	0.6312	-0.3895	0.0106	0.0312	0.6934	0.9523	0.9109	0.8918	1.0000
Buena Vista (BVA)												
pH	1.0000	0.4727	0.4142	0.1493	-0.5684	-0.1202	-0.2784	-0.2500	-0.0645	-0.2370	-0.3571	-0.0748
Phosphate	0.4727	1.0000	-0.2895	-0.2556	-0.2537	0.2779	-0.0417	0.5263	0.2637	-0.2523	-0.5575	0.3445
Nitrite	0.4142	-0.2895	1.0000	0.0650	-0.2061	-0.3836	0.0358	-0.7433	-0.3041	0.1434	0.2857	-0.3409
Nitrate	0.1493	-0.2556	0.0650	1.0000	-0.1896	-0.0061	0.4202	-0.0995	0.2631	0.5589	0.6293	0.2669
Ammonium	-0.5684	-0.2537	-0.2061	-0.1896	1.0000	-0.4134	-0.1979	0.1439	-0.4331	-0.3100	-0.2903	-0.4689
Sulfate	-0.1202	0.2779	-0.3836	-0.0061	-0.4134	1.0000	-0.0491	0.3826	0.3345	-0.3075	-0.1694	0.3492
Methanobacteriaceae	-0.2784	-0.0417	0.0358	0.4202	-0.1979	-0.0491	1.0000	-0.2560	0.7044	0.8408	0.7744	0.6608
Methanoregulaceae	-0.2500	0.5263	-0.7433	-0.0995	0.1439	0.3826	-0.2560	1.0000	0.1582	-0.3427	-0.3395	0.1766
Methanosaetaceae	-0.0645	0.2637	-0.3041	0.2631	-0.4331	0.3345	0.7044	0.1582	1.0000	0.4764	0.3952	0.8377
Methanosarcinaceae	-0.2370	-0.2523	0.1434	0.5589	-0.3100	-0.3075	0.8408	-0.3427	0.4764	1.0000	0.8553	0.5254
Methanocellaceae	-0.3571	-0.5575	0.2857	0.6293	-0.2903	-0.1694	0.7744	-0.3395	0.3952	0.8553	1.0000	0.3838
Methanomassiliicoccaceae	-0.0748	0.3445	-0.3409	0.2669	-0.4689	0.3492	0.6608	0.1766	0.8377	0.5254	0.3838	1.0000

3 Supplementary References

Artz, R., Chapman, S., and Campbell, C. (2006). Substrate utilisation profiles of microbial communities in peat are depth dependent and correlate with whole soil FTIR profiles. *Soil Biology and Biochemistry* 38, 2958–2962. doi:10.1016/j.soilbio.2006.04.017.

Bai, Y., Wang, J., Zhan, Z., Guan, L., Jin, L., Zheng, G., et al. (2018). The variation of microbial communities in a depth profile of peat in the Gahai Lake wetland natural conservation area. *Geomicrobiology Journal* 35, 484–490. doi:10.1080/01490451.2017.1392651.

Barta, J., Tahovska, K., Santruckova, H., and Oulehle, F. (2017). Microbial communities with distinct denitrification potential in spruce and beech soils differing in nitrate leaching. *Sci. Rep.* 7, 1–15. doi:DOI:10.1038/s41598-017-08554-1.

Beneduzi, A., Costa, P. B., Parma, M., Melo, I. S., Bodanese-Zanettini, M. H., and Passaglia, L. M. P. (2010). *Paenibacillus riograndensis* sp. nov., a nitrogen-fixing species isolated from the rhizosphere of *Triticum aestivum*. *International Journal Of Systematic And Evolutionary Microbiology* 60, 128–133. doi:10.1099/ijs.0.011973-0.

Bondoso, J., Albuquerque, L., Lobo-da-Cunha, A., da Costa, M. S., Harder, J., and Lage, O. M. (2014). *Rhodopirellula lusitana* sp. nov. and *Rhodopirellula rubra* sp. nov., isolated from the surface of macroalgae. *Systematic and Applied Microbiology* 37, 157–164. doi:10.1016/j.syapm.2013.11.004.

Chin, K.-J., Liesack, W., and Janssen, P. H. (2001). *Opitutus terrae* gen. nov., sp. nov., to accommodate novel strains of the division "*Verrucomicrobia*" isolated from rice paddy soil. *International Journal of Systematic Bacteriology* 51, 1965–1968.

Eichorst, S. A., Kuske, C. R., and Schmidt, T. M. (2011). Influence of plant polymers on the distribution and cultivation of bacteria in the phylum *Acidobacteria*. *Appl. Environ. Microbiol.* 77, 586–596. doi:10.1128/AEM.01080-10.

Evans, P. N., Parks, D. H., Chadwick, G. L., Robbins, S. J., Orphan, V. J., Golding, S. D., et al. (2015). Methane metabolism in the archaeal phylum Bathyarchaeota revealed by genome-centric metagenomics. *Science* 350, 434–438.

Florentino, A. P., Stams, A. J. M., and Sánchez-Andrea, I. (2017). Genome sequence of *Desulfurella amilsii* strain TR1 and comparative genomics of *Desulfurellaceae* Family. *Front. Microbiol.* 8, k39. doi:10.3389/fmicb.2017.00222.

Gandois, L., Teisserenc, R., Cobb, A. R., Chieng, H. I., Lim, L. B. L., Kamariah, A. S., et al. (2014). Origin, composition, and transformation of dissolved organic matter in tropical peatlands. *Geochimica et Cosmochimica Acta* 137, 35–47. doi:10.1016/j.gca.2014.03.012.

Heitmann, T., Goldhammer, T., Beer, J., and Blodau, C. (2007). Electron transfer of dissolved organic matter and its potential significance for anaerobic respiration in a northern bog. *Glob Change Biol* 13, 1771–1785. doi:10.1111/j.1365-2486.2007.01382.x.

Honório, B. A. D., Horbe, A. M. C., and Seyler, P. (2010). Chemical composition of rainwater in western Amazonia — Brazil. *Atmospheric Research* 98, 416–425.

doi:10.1016/j.atmosres.2010.08.001.

Khramenkov, S. V., Kozlov, M. N., Kevbrina, M. V., Dorofeev, A. G., Kazakova, E. A., Grachev, V. A., et al. (2013). A novel bacterium carrying out anaerobic ammonium oxidation in a reactor for biological treatment of the filtrate of wastewater fermented sludge. *Microbiology* 82, 628–636. doi:10.1134/S002626171305007X.

Knorr, K.-H., and Blodau, C. (2009). Impact of experimental drought and rewetting on redox transformations and methanogenesis in mesocosms of a northern fen soil. *Soil Biology and Biochemistry* 41, 1–12. doi:10.1016/j.soilbio.2009.02.030.

Lazar, C. S., Baker, B. J., Seitz, K., Hyde, A. S., Dick, G. J., Hinrichs, K.-U., et al. (2016). Genomic evidence for distinct carbon substrate preferences and ecological niches of Bathyarchaeota in estuarine sediments. *Environmental Microbiology* 18, 1200–1211.

Lähteenoja, O., Ruokolainen, K., Schulman, L., and Alvarez, J. (2009). Amazonian floodplains harbour minerotrophic and ombrotrophic peatlands. *Catena* 79, 140–145. doi:10.1016/j.catena.2009.06.006.

Liu, D., Ding, W., Jia, Z., and Cai, Z. (2012). The impact of dissolved organic carbon on the spatial variability of methanogenic archaea communities in natural wetland ecosystems across China. *Appl Microbiol Biotechnol* 96, 253–263. doi:10.1007/s00253-011-3842-x.

Lueders, T., Wagner, B., Claus, P., and Friedrich, M. W. (2003). Stable isotope probing of rRNA and DNA reveals a dynamic methylotroph community and trophic interactions with fungi and protozoa in oxic rice field soil. *Environmental Microbiology* 6, 60–72. doi:10.1046/j.1462-2920.2003.00535.x.

Mandić-Mulec, I., Stefanic, P., and van Elsas, J. D. (2015). Ecology of *Bacillaceae*. *Microbiology Spectrum* 3, 1–24. doi:10.1128/microbiolspec.TBS-0017-2013.

Martineau, C., Mauffrey, F., and Villemur, R. (2015). Comparative analysis of denitrifying activities of *Hyphomicrobium nitrativorans, Hyphomicrobium denitrificans*, and *Hyphomicrobium zavarzinii*. *Appl. Environ. Microbiol.* 81, 5003–5014. doi:10.1128/AEM.00848-15.

Miroshnichenko, M. L., Rainey, F. A., Hippe, H., Chernyh, N. A., and Bonch-Osmolovskaya, E. A. (1998). *Desulfurella kamchatkensis* sp. nov. and *Desulfurella propionica* sp. nov., new sulfur-respiring thermophilic bacteria from Kamchatka thermal environments. *International Journal of Systematic Bacteriology* 48, 475–479.

Moers, M. E. C., Baas, M., Boon, J. J., and De Leeuw, J. W. (1990). Molecular characterization of total organic matter and carbohydrates in peat samples from a cypress swamp by pyrolysis-mass spectrometry and wet-chemical methods. *Biogeochemistry* 11, 251–277. doi:10.1007/BF00004499.

Mohagheghi, A., Grohmann, K., Himmel, M., Leighton, L., and Updegraff, D. M. (1986). Isolation and characterization of *Acidothermus cellulolyticus* gen. nov., sp. nov, a new genus of thermophilic, acidophilic, cellulolytic bacteria. *International Journal of Systematic Bacteriology* 36, 435–443.

Morrissey, E. M., Berrier, D. J., Neubauer, S. C., and Franklin, R. B. (2013). Using microbial communities and extracellular enzymes to link soil organic matter characteristics to greenhouse gas production in a tidal freshwater wetland. *Biogeochemistry* 117, 473–490. doi:10.1007/s10533-013-9894-5.

Rock, J. D., Mahnane, M. R., Anjum, M. F., Shaw, J. G., Read, R. C., and Moir, J. W. B. (2005). The pathogen Neisseria meningitidisrequires oxygen, but supplements growth by denitrification. Nitrite, nitric oxide and oxygen control respiratory flux at genetic and metabolic levels. *Molecular Microbiology* 58, 800–809. doi:10.1111/j.1365-2958.2005.04866.x.

Rosenberg, E., DeLong, E. F., Lory, S., Stackebrandt, E., and Thompson, F. eds. (2014). *The Prokaryotes*. Heidelberg, New York, Dordrecht, London, 4th Edition: Springer-Verlag.

Troshina, O., Oshurkova, V., Suzina, N., Novikov, A., Shcherbakova, V., Vinokurova, N., et al. (2015). *Sphaerochaeta associata* sp. nov., a spherical spirochaete isolated from cultures of *Methanosarcina mazei* JL01. *International Journal Of Systematic And Evolutionary Microbiology* 65, 4315–4322. doi:10.1099/ijsem.0.000575.

Vaz-Moreira, I., Figueira, V., Lopes, A. R., De Brandt, E., Vandamme, P., Nunes, O. C., et al. (2011). *Candidimonas nitroreducens* gen. nov., sp. nov. and *Candidimonas humi* sp. nov., isolated from sewage sludge compost. *International Journal Of Systematic And Evolutionary Microbiology* 61, 2238–2246. doi:10.1099/ijs.0.021188-0.

Yang, S., Liebner, S., Winkel, M., Alawi, M., Horn, F., Dörfer, C., et al. (2017). In-depth analysis of core methanogenic communities from high elevation permafrost-affected wetlands. *Soil Biology and Biochemistry* 111, 66–77. doi:10.1016/j.soilbio.2017.03.007.

Yoon, J. H. (2003). *Paenibacillus kribbensis* sp. nov. and *Paenibacillus terrae* sp. nov., bioflocculants for efficient harvesting of algal cells. *International Journal Of Systematic And Evolutionary Microbiology* 53, 295–301. doi:10.1099/ijs.0.02108-0.

Zahran, H. H. (1999). *Rhizobium*-legume symbiosis and nitrogen fixation under severe conditions and in an arid climate. *Microbiology and Molecular Biology Reviews* 63, 968–989.

Zaichikova, M. V., Berestovskaya, Y. Y., Akimov, V. N., Kizilova, A. K., and Vasilieva, L. V. (2010). *Xanthobacter xylophilus* sp. nov., a member of the xylotrophic mycobacterial community of low-mineral oligotrophic waters. *Microbiology* 79, 83–88. doi:10.1134/S002626171001011X.
